# Predicting Mortality After Percutaneous Coronary Intervention in a Multiethnic Southeast Asian Population: Insights From Machine Learning

**DOI:** 10.1007/s12265-026-10812-5

**Published:** 2026-07-06

**Authors:** Yih Miin Liew, Yin Kia Chiam, Pei Ling Ngo, Hui Yee Tan, Nor Ashikin Md Sari, Li Kuo Tan, Wan Azman Wan Ahmad, Kok Han Chee

**Affiliations:** 1 Department of Biomedical Engineering, Faculty of Engineering, Universiti Malaya, Kuala Lumpur, 50603 Malaysia; 2 Department of Software Engineering, Faculty of Computer Science and Information Technology, Universiti Malaya, Kuala Lumpur, 50603 Malaysia; 3 Department of Medicine, University Malaya Medical Centre, Kuala Lumpur, 50603 Malaysia; 4 Department of Biomedical Imaging, Faculty of Medicine, Universiti Malaya, Kuala Lumpur, Malaysia; 5 University Malaya Research Imaging Centre, Universiti Malaya, Kuala Lumpur, Malaysia

**Keywords:** Percutaneous coronary intervention, Machine learning, Patient mortality prediction, Risk factors analysis, Cardiovascular disease

## Abstract

Ischemic heart disease remains a major contributor to mortality in Malaysia, with non-elective percutaneous coronary intervention (PCI) frequently performed in high-risk acute coronary syndrome (ACS) patients. Using nationwide registry data (2007–2020), we evaluated 29,521 patients and compared seven machine learning (ML) models for predicting in-hospital, 30-day, and 1-year mortality. Models were developed in a training cohort and externally validated using hospital-level (TEST1) and prospective temporal (TEST2) cohorts. After logistic recalibration, discrimination for in-hospital mortality ranged from 0.927 to 0.943 (TEST1) and 0.865–0.884 (TEST2). For 30-day mortality, ROC-AUC ranged from 0.902 to 0.923 (TEST1) and 0.753–0.838 (TEST2), and for 1-year mortality from 0.833 to 0.859 (TEST1) and 0.750–0.801 (TEST2). Calibration remained acceptable, and decision curve analysis demonstrated positive net benefit across clinically relevant thresholds. Cross-model stability analysis consistently identified age, haemodynamic status, and renal function as key predictors.

**Supplementary Information:**

The online version contains supplementary material available at 10.1007/s12265-026-10812-5.

## Introduction

Ischemic heart disease remains a leading cause of morbidity and mortality worldwide and is the principal cause of certified deaths in Malaysia, accounting for 15.1% of all deaths in 2023 [1]. Percutaneous coronary intervention (PCI) is central to the management of acute coronary syndrome (ACS), particularly in high-risk presentations. Over the past two decades, PCI utilisation in Malaysia has expanded steadily, as documented in the Malaysian National Cardiovascular Disease-PCI (NCVD-PCI) registry. Notably, the relatively young mean age of PCI recipients (57.8 years) underscores the substantial socioeconomic burden of premature cardiovascular disease in this population [2].

Accurate mortality risk stratification following PCI is critical for clinical triage, discharge planning, and secondary prevention. Established predictors include advanced age, haemodynamic instability, renal dysfunction, left ventricular impairment, and lesion complexity [3]. However, most contemporary risk models have been developed in Western cohorts and frequently rely on traditional regression-based approaches [4, 5]. Differences in case mix, healthcare systems, and ethnic composition may limit the transportability of these models to multiethnic Southeast Asian populations. Moreover, model performance is often reported using internal validation alone, which may overestimate generalisability.

Machine learning (ML) methods provide flexible modelling frameworks capable of capturing non-linear effects and higher-order interactions without prespecified functional forms [6]. While ML has shown promise in cardiovascular risk prediction, improvements over well-specified regression models are not universal, and performance may vary across settings. Importantly, discrimination alone is insufficient for clinical implementation; calibration stability and external validation across independent cohorts are essential to ensure reliable risk estimation. Given increasing scrutiny regarding reproducibility and model transportability in clinical AI, such rigorous validation is critical for meaningful translational impact.

Analyses of the NCVD-PCI registry have previously focused on selected subgroups, such as young cohorts, renal impairment, or access-site comparisons, primarily using conventional statistical methods [7, 8]. To date, only one prior ML study has used NCVD-PCI data, and it was restricted to a single centre [9]. A comprehensive multicentre evaluation comparing multiple modelling approaches, with rigorous geographical and temporal validation, remains lacking.

In this context, the present study leverages nationwide NCVD-PCI registry data (2007–2020) to (1) evaluate in-hospital, 30-day, and 1-year mortality following non-elective PCI for ACS; (2) compare the predictive performance and calibration of seven ML algorithms within a unified framework; and (3) identify clinically stable predictors using cross-model importance analysis. By incorporating hospital-level and prospective temporal validation, this study aims to assess not only model discrimination but also transportability and clinical utility in a real-world, multiethnic Southeast Asian population.

## Materials and Methods

### Dataset and Registry Description

This study utilised data from the Malaysian NCVD-PCI Registry (2007–2020), which prospectively collects standardized demographic, clinical, procedural, and outcome data from public and private PCI centres nationwide. Data quality is maintained through structured case report forms, trained data collectors, centralized electronic entry with built-in validation rules, and periodic audits [2]. Mortality status is routinely cross-verified against the Malaysian National Registration Department.

### Data Harmonization and Structural Consolidation

Registry data were collected across five phases (2007–2012, 2013–2014, 2015–2016, 2017–2018, and 2019–2020), requiring cross-era harmonisation. Cohort-specific Excel files (patient, lesion, procedure, stent) were converted to columnar Parquet format for computational efficiency. Variable names and coding schemes were standardized using predefined mapping tables. Date formats were harmonized, registry-specific sentinel codes were recoded as missing, and exact duplicates were removed. After cross-table merging for patient-level linkage, 93,451 unique patients were available for eligibility assessment.

### Study Population and Cohort Derivation

Eligibility criteria were applied sequentially. We included non-elective PCI for ACS (STEMI, NSTEMI, unstable angina) and excluded elective procedures, chronic stable angina, non-Malaysians, age < 18 years, missing lesion identifiers, or missing mortality outcomes. Only the first admission and first PCI per patient were retained.

### Feature Engineering and Final Predictor Definition

Lesion-level records were aggregated to patient level using predefined rule-based strategies developed with interventional cardiology input. Aggregation included first non-missing value for categorical variables, logical OR for binary indicators, minimum for worst physiological measures (e.g., lowest TIMI flow), and maximum, mean, or sum for continuous variables as clinically appropriate.

Derived variables included number of treated vessels, multivessel PCI indicator, total lesion length, count of long lesions (> 20 mm), lesion number, and stent characteristics. Continuous variables were assessed against physiology-based plausibility ranges derived from NCVD reports and expert consensus; implausible values were set to missing without distribution-based trimming. All continuous predictors were retained in continuous form without discretization, and these ranges were applied solely for quality control. Ethnicity was consolidated a priori from 12 registry categories into four groups (Malay, Chinese, Indian, Others) to minimise sparse-category instability, independent of outcome status. Variables with > 50% missingness and patients with > 50% missing predictor data were excluded a priori.

**Fig. 1:**
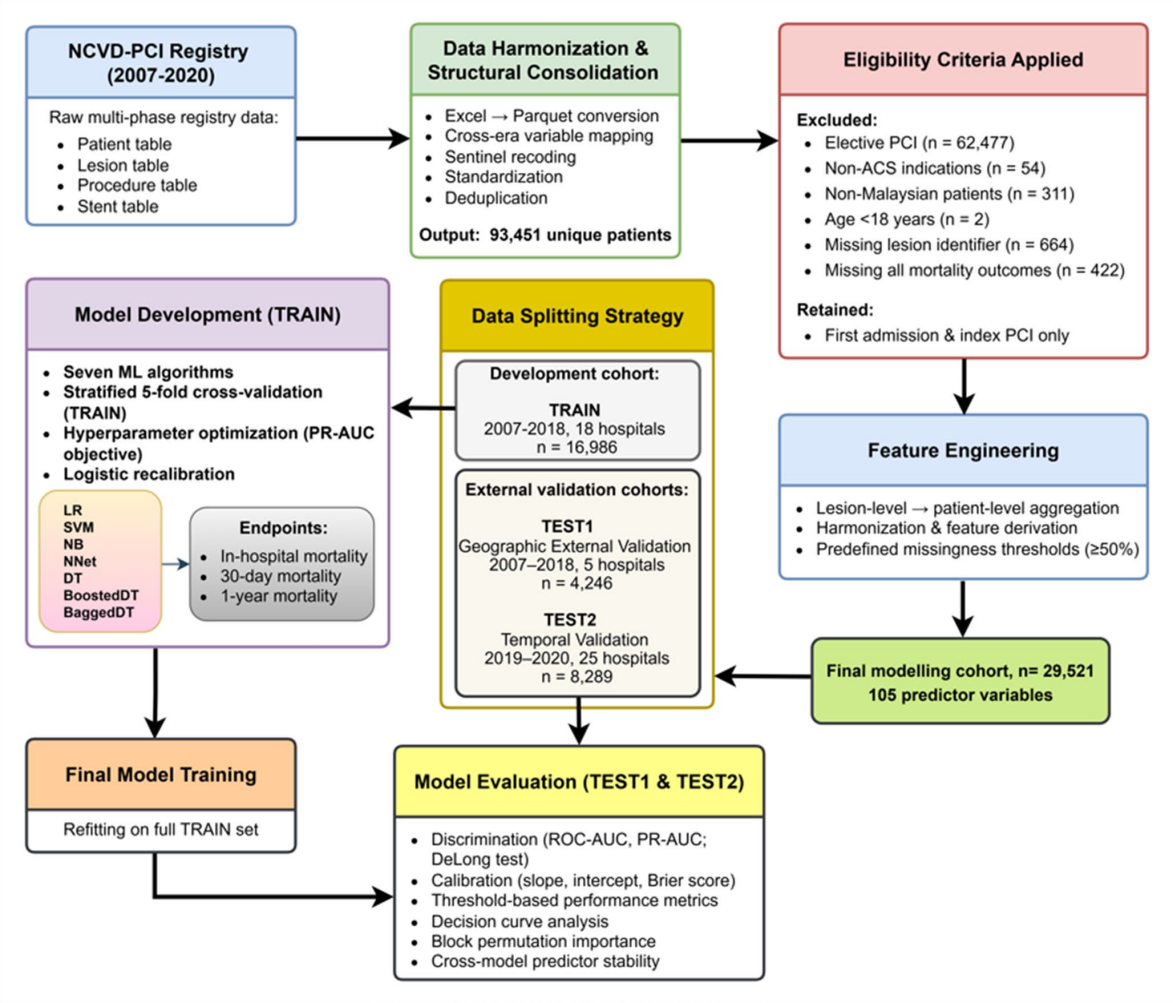
Analytical workflow for model development and external validation using NCVD-PCI registry data (2007–2020)

The final analytic dataset comprised 29,521 patients with 105 clinically curated predictor variables spanning demographics, comorbidities, cardiac status, and PCI characteristics. Predictor inclusion was determined a priori based on clinical relevance and availability across registry eras, independent of outcome status. Risk prediction was defined at completion of the index PCI procedure, using only variables available at or prior to that timepoint. Full predictor definitions are provided in the Supplementary Table A1. All harmonisation, aggregation, and feature derivation steps were completed prior to dataset splitting to ensure identical predictor definitions across model development and external validation cohorts. Figure 1 illustrates the overall analytical workflow from registry extraction to model evaluation.

### Outcome Definition

The primary endpoints were all-cause mortality at three clinically relevant time points: in-hospital, 30-day, and 1-year mortality following index PCI. Mortality was confirmed through registry documentation and cross-referenced with the Malaysian National Registration Department. Patients with missing follow-up at a specific time point were excluded from analyses for that endpoint only, without assuming survival. Each endpoint was analysed separately using only patients with documented follow-up. No imputation of survival status was performed.

### Data Splitting Strategy

Data were partitioned using a combined hospital-based and temporal strategy (Table 1). Patients treated between 2007 and 2018 from 18 hospitals (*n* = 16,986) formed the TRAIN cohort for model development and internal cross-validation. Five hospitals from the same period (*n* = 4,246) were held out as geographically independent TEST1. All eligible patients from 2019 to 2020 across 25 hospitals (*n* = 8,289) formed temporally independent TEST2. Hospitals contributing to TEST1 were excluded entirely from model development. The 2019–2020 cohort was held out to evaluate prospective temporal generalisability. Detailed hospital-level allocation for model development and external validation is provided in Table A2 (Supplementary Material).

**Table 1:** Hospital-based and temporal data split for model development and validation

Dataset	Cohort years	Split type	Number of hospitals	Number of patients	Purpose
Training + internal CV (TRAIN)	2007–2018	Hospital-based	18	16,986	Model development, hyperparameter tuning, threshold selection
External validation (TEST1)	2007–2018	Hospital-based (held-out hospitals)	5	4,246	External validation (geographical generalisability)
External validation (TEST2)	2019–2020	Temporal split	25	8,289	Temporal validation (prospective generalisability)
Total	**2007–2020**	—	**25**	**29**,**521**	—

### Model Development

All models were developed exclusively using the TRAIN cohort. TEST1 and TEST2 were reserved for external validation and were not accessed during preprocessing, feature selection, hyperparameter tuning, calibration, or threshold selection. Separate models were trained for in-hospital, 30-day, and 1-year mortality. Seven supervised ML algorithms were evaluated within a unified preprocessing and validation framework: LASSO-regularized logistic regression (LR), support vector machine (SVM), Naïve Bayes (NB), feedforward neural network (NNet), decision tree (DT), bagged decision trees (BaggedDT), and boosted decision trees (BoostedDT). These seven algorithms were selected to cover a spectrum of commonly used clinical prediction approaches with different modelling assumptions and levels of algorithmic complexity: a regularised linear baseline (LR), margin-based/kernel learning (SVM), probabilistic classification (NB), nonlinear neural network modelling (NNet), an interpretable single-tree model (DT), and two ensemble tree methods designed to reduce variance (BaggedDT) or sequentially correct errors (BoostedDT). Within this framework, the single decision tree was intentionally retained as an interpretable, low-complexity benchmark rather than as an expected top-performing model, establishing a structural baseline for rule-based partitioning.

#### Predictor Preprocessing

Predictors were specified a priori and categorised as continuous, ordinal, binary, or nominal. Clinically ordered variables (e.g., NYHA, Killip, CCS class, TIMI flow) were encoded numerically to preserve ordinal structure. Binary variables were converted to numeric format (0/1). Missing data handling was performed before categorical encoding: continuous variables were imputed using median imputation, which was selected to ensure consistent preprocessing across algorithms and to minimise the risk of information leakage or outcome-informed imputation; categorical variables included an explicit “Missing” category; nominal predictors were one-hot encoded using category levels defined strictly from TRAIN. The encoding structure was fixed and applied unchanged to TEST1 and TEST2. Continuous predictors were standardised where required (e.g., support vector machine, neural network) using TRAIN-derived mean and standard deviation.

#### Model Implementation and Hyperparameter Specification

Implementation details and hyperparameter specifications for each modelling approach are outlined below.

##### LASSO-regularized Logistic Regression (LR)

LR with L1 regularisation was implemented. The penalty parameter (λ) was selected via cross-validation over a logarithmic grid. Predictors with |β|>10⁻⁶ were considered selected.

##### Support Vector Machine (SVM)

Linear and radial basis function (RBF) kernels were implemented using the Sequential Minimal Optimization solver. Tuned hyperparameters included the box constraint (C) and kernel scale (for RBF).

##### Naïve Bayes (NB)

NB was implemented in Gaussian-multinomial (“mixed”) and kernel density modes. Hyperparameters included modelling mode, class prior, and kernel bandwidth. To mitigate redundancy under the conditional independence assumption, minimum redundancy maximum relevance (mRMR) feature selection was performed within each cross-validation training fold, with the number of retained predictors (K) optimized during cross-validation. Predictors with zero within-class variance were excluded.

##### Feedforward Neural Network (NNet)

Fully connected neural networks were trained using cross-entropy loss. Tuned hyperparameters included hidden layer architecture, activation function (ReLU or tanh), L2 regularisation strength (λ), and maximum iteration limits. Continuous predictors were standardised using TRAIN-derived statistics.

##### Decision Tree (DT)

Classification trees were trained using recursive binary partitioning. Tuned hyperparameters included maximum number of splits, minimum leaf size, and split criterion (Gini impurity or cross-entropy). This model was included as a reference comparator for interpretability and to contextualise the performance gain achieved by ensemble tree methods.

##### Bagged Decision Trees (BaggedDT)

Bootstrap-aggregated trees (random forest variant) were trained, with probabilities averaged across trees. Tuned parameters included number of trees, maximum splits, minimum leaf size, and feature subsampling per split (“all” or √p).

##### Boosted Decision Trees (BoostedDT)

Ensembles of shallow trees were trained sequentially using boosting algorithms (LogitBoost, GentleBoost, AdaBoostM1, and RUSBoost). Tuned hyperparameters included number of learning cycles, learning rate, maximum splits, and minimum leaf size.

Regularisation parameters controlled model complexity without separate univariate pre-filtering. The complete hyperparameter search ranges and selected optimal configurations are provided in Supplementary Material (Table A3).

#### Cross***-***Validation and Hyperparameter Optimization

Model development employed stratified 5-fold cross-validation within the TRAIN cohort. Within each fold, preprocessing (imputation, encoding, scaling, and fold-specific feature selection where applicable) was estimated using training-fold data only and applied to the corresponding validation fold to prevent information leakage. Out-of-fold (OOF) predicted probabilities were obtained by concatenating validation-fold predictions across folds, ensuring that each observation was evaluated using a model trained on disjoint data.

Given the low event prevalence, inverse-frequency class weighting (normalized to a mean of 1) was applied during model fitting within TRAIN folds only. No imbalance correction was applied to TEST1 or TEST2. Synthetic resampling methods (e.g., SMOTE or random undersampling) were not used to preserve the natural outcome distribution and maintain reliable probability estimates in external validation.

Hyperparameters were optimized using grid search, with mean OOF precision-recall AUC (PR-AUC) as the selection criterion. The search space comprised 60 configurations for LR, 52 for SVM, 120 for NB, 36 for NNet, 32 for DT, 54 for BaggedDT, and 192 for BoostedDT.

#### Probability Calibration

After hyperparameter selection, logistic recalibration (Platt-style) was fitted using TRAIN OOF predictions:

$$\:\mathrm{logit}\left({p}_{cal}\right)=\alpha\:+\beta\:\cdot\:\mathrm{logit}\left({p}_{raw}\right)$$

For SVM, the raw decision score was used in place of $\:\mathrm{logit}\left({p}_{raw}\right)$. The calibration parameters (α, β) were estimated exclusively from TRAIN and fixed prior to external validation.

#### Final Refit and Threshold Determination

Using the selected hyperparameters, each model was retrained on the full TRAIN dataset. Calibrated probabilities for the refitted models were obtained using previously estimated calibration parameters (*α*, *β*). A single operating threshold was derived from calibrated TRAIN predictions using the Youden Index and applied unchanged to TEST1 and TEST2.

### Model Evaluation, Clinical Utility and Interpretability

Discrimination was evaluated using ROC-AUC and PR-AUC with 95% confidence intervals estimated using nonparametric bootstrap resampling. Pairwise model comparisons in external validation datasets were performed using DeLong’s test.

Calibration was assessed using calibration slope, calibration intercept, and Brier score. Calibration plots were constructed using decile-based risk grouping. Clinical utility was evaluated using decision curve analysis across threshold probabilities of 1–50%, comparing net benefit against treat-all and treat-none strategies.

Interpretability was assessed using block-level permutation importance on TRAIN following final model refitting. Encoded variables representing each clinical construct were jointly permuted, and mean ROC-AUC reduction across five permutation repeats was computed. Importance was converted to within-model ranks (most important = highest AUC drop), which were subsequently normalised to [0,1] to enable cross-model comparison. This block-level approach mitigates artificial inflation of importance arising from one-hot encoding and preserves interpretability at the clinical construct level.

Predictor stability across algorithms was summarised using the median rank and IQR across models. Predictors were considered stable if they ranked within the top 30 in at least five of seven models. Stability was evaluated separately for in-hospital, 30-day, and 1-year mortality to identify predictors demonstrating consistent importance across time horizons.

All analyses were conducted in MATLAB (R2024b) with fixed random seeds. GPU acceleration was used where supported. Software environment details are summarised in Supplementary Table A4. Survival modelling was not performed, as the objective was fixed-horizon risk prediction.

## Results

### Analytic Cohort and Mortality Prevalence

A total of 29,521 patients met eligibility criteria and were included in the analysis, comprising 16,986 patients in TRAIN (2007–2018), 4,246 in TEST1 (hospital hold-out), and 8,289 in TEST2 (2019–2020 temporal validation).

Mortality prevalence among patients with documented follow-up is shown in Table 2. In-hospital mortality was 5.14% in TRAIN, 5.51% in TEST1, and 3.18% in TEST2. Thirty-day mortality was 6.82%, 7.62%, and 4.84%, respectively. One-year mortality was 11.42%, 11.96%, and 9.21%. In-hospital follow-up was complete. Missing follow-up at 30 days and 1 year occurred mainly in earlier registry years, particularly within TRAIN (5.0% and 7.1%). Mortality was consistently lower in the temporal cohort (2019–2020).

**Table 2:** Outcome prevalence across training and external validation cohorts

Outcome	Cohort	*N*	Follow-up	Missing	Deaths, *n* (%)	Alive, *n* (%)
In-hospital	TRAIN	16,986	16,986	0	873 (5.14)	16,113 (94.86)
	TEST1	4,246	4,246	0	234 (5.51)	4,012 (94.49)
	TEST2	8,289	8,289	0	264 (3.18)	8,025 (96.82)
30-day	TRAIN	16,986	16,133	853	1,100 (6.82)	15,033 (93.18)
	TEST1	4,246	3,832	414	292 (7.62)	3,540 (92.38)
	TEST2	8,289	8,282	7	401 (4.84)	7,881 (95.16)
1-year	TRAIN	16,986	15,780	1206	1,802 (11.42)	13,978 (88.58)
	TEST1	4,246	3,688	558	441 (11.96)	3,247 (88.04)
	TEST2	8,289	8,281	8	763 (9.21)	7,518 (90.79)

Patient Demographics and Baseline Characteristics

Values are reported as n (%) among patients with documented follow-up

Baseline characteristics are presented in Supplementary Table A5. Median age was 56.7 years (IQR 48.9–64.4), and 84.3% were male, with no significant differences across cohorts (*p* > 0.80; standardized mean difference (SMD) < 0.01). Ethnic distribution differed (*p* < 0.001), with greater variation in TEST1 (SMD = 0.327), whereas TEST2 closely resembled TRAIN (SMD = 0.089).

Cardiometabolic risk factors were common: hypertension (63.2%), diabetes (43.2%), and dyslipidaemia (51.7%). Although several variables reached statistical significance, most SMDs were < 0.20, indicating small effect sizes; the largest imbalance was dyslipidaemia in TEST1 (SMD = 0.226).

Hemodynamic and renal markers showed minimal imbalance (SMD ≤ 0.19 and < 0.10, respectively). STEMI predominated overall (65.1%), with lower prevalence in TEST2 (59.1%) compared with TRAIN (67.3%) and TEST1 (68.5%) (SMD = 0.191). No large imbalances (SMD > 0.50) were observed.

### Model Discrimination

**Fig. 2:**
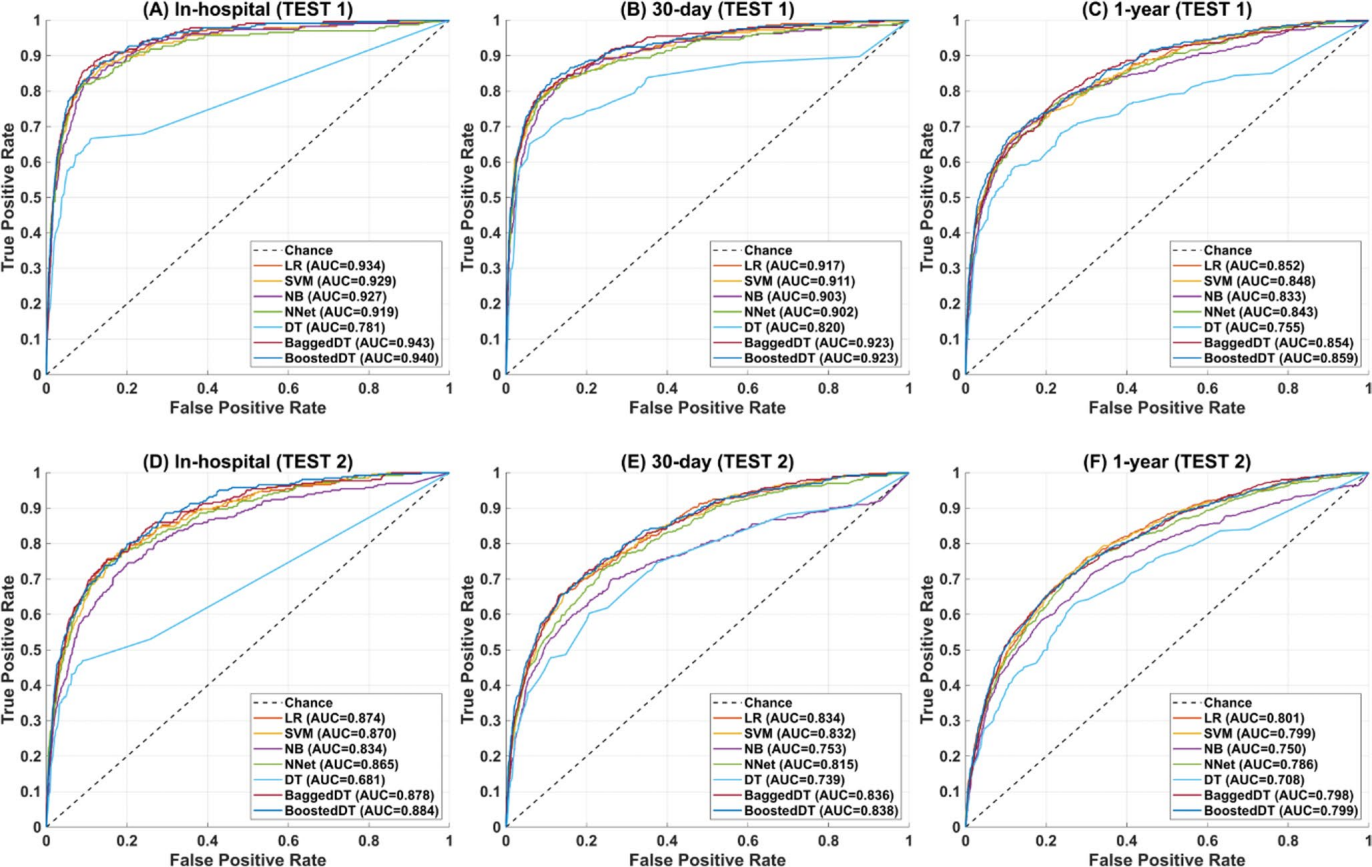
ROC curves for in-hospital, 30-day, and 1-year mortality in external hospital validation (TEST1; A-C) and temporal validation (TEST2; D-F)

Discrimination was evaluated using ROC-AUC and PR-AUC in TEST1 and TEST2 (Fig. 2; Supplementary Table A6).

For in-hospital mortality, discrimination was strong for most models in TEST1 (ROC-AUC 0.919–0.943), with ensemble tree-based models achieving the highest AUCs (BaggedDT 0.943; BoostedDT 0.940), closely followed by LR (0.934). The single DT performed lower (ROC-AUC 0.781). PR-AUC ranged from 0.545 to 0.585 in TEST1 (event prevalence 5.51%), substantially exceeding baseline event probability. In TEST2, performance attenuated modestly (ROC-AUC 0.865–0.884), and PR-AUC ranged from 0.301 to 0.318 (event prevalence 3.18%), reflecting lower outcome prevalence rather than deterioration in ranking performance.

For 30-day mortality, discrimination remained high for most models in TEST1 (ROC-AUC 0.902–0.923). In TEST2, ROC-AUC ranged from 0.753 to 0.838, demonstrating modest temporal attenuation with preserved model ordering. PR-AUC ranged from 0.608 to 0.646 in TEST1 (prevalence 7.62%) and 0.293–0.320 in TEST2 (prevalence 4.84%), again remaining substantially above baseline rates.

For 1-year mortality, discrimination was lower overall. ROC-AUC ranged from 0.833 to 0.859 in TEST1 and 0.750–0.801 in TEST2 among most models, with LR achieving the highest temporal AUC (0.801). PR-AUC ranged from 0.530 to 0.590 in TEST1 (prevalence 11.96%) and 0.289–0.361 in TEST2 (prevalence 9.21%).

Pairwise DeLong comparisons (Supplementary Table A7) indicated that although some differences were statistically significant, absolute AUC differences between LR and ensemble models were small (< 0.05), and no model demonstrated consistent clinically meaningful superiority across endpoints and validation cohorts.

### Calibration Performance

**Fig. 3:**
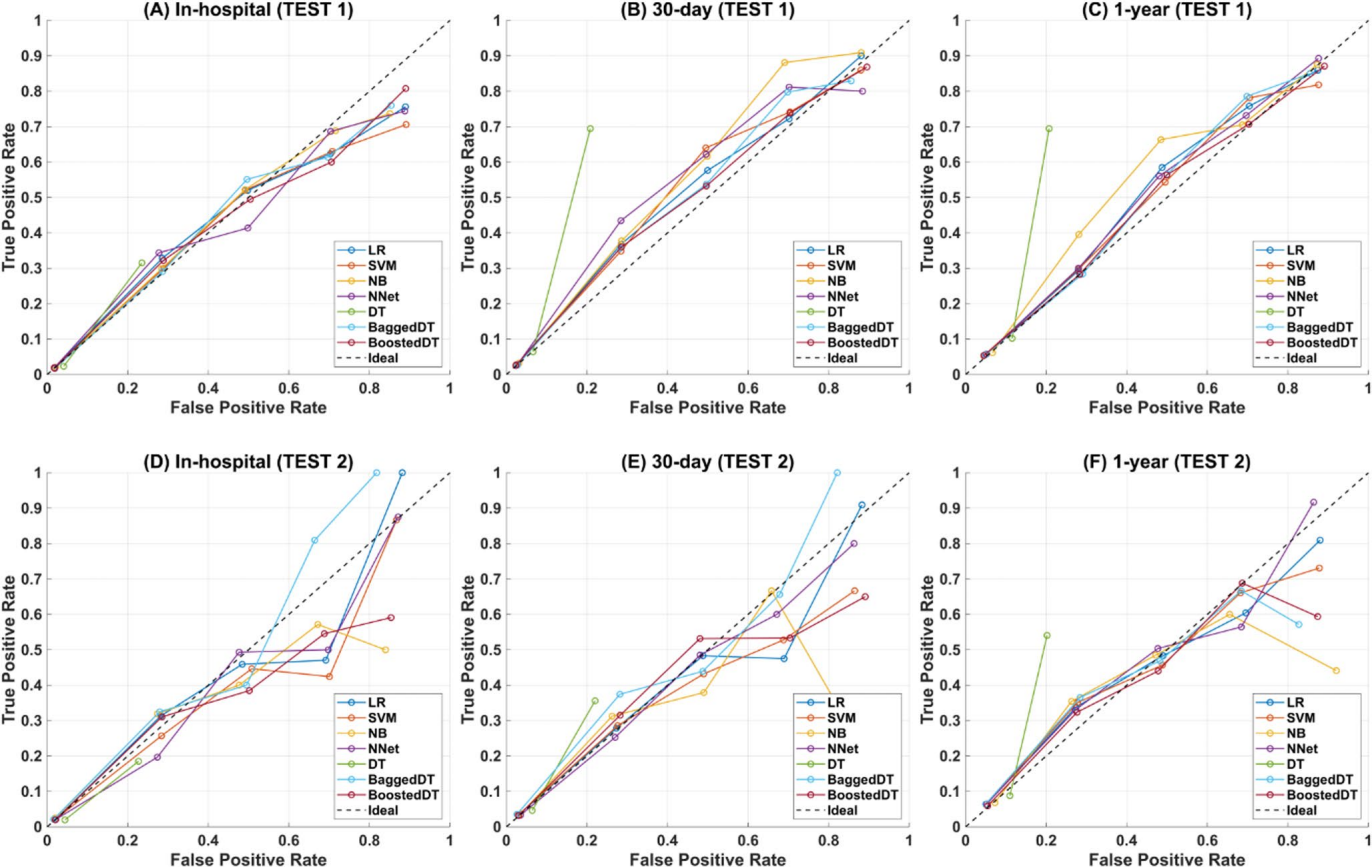
Calibration plots for in-hospital, 30-day, and 1-year mortality in TEST1 (**A**-**C**) and TEST2 (**D**-**F**). Points represent decile-based observed versus predicted risk; the dashed line indicates perfect calibration

Calibration metrics are shown in Supplementary Table A8 and Fig. 3. Across endpoints, Brier scores were low and comparable across models. For in-hospital mortality, Brier scores ranged from 0.032 to 0.045 in TEST1 and 0.026–0.031 in TEST2. For 30-day mortality, scores ranged from 0.040 to 0.063 in TEST1 and 0.039–0.044 in TEST2, while for 1-year mortality they ranged from 0.071 to 0.099 in TEST1 and 0.071–0.081 in TEST2. Brier scores increased with longer follow-up, consistent with greater uncertainty in long-term prediction.

In TEST1, LR and ensemble models showed intercepts near zero and slopes near unity, indicating good agreement between predicted and observed risk. In contrast, NB and DT exhibited greater instability, particularly for 30-day mortality, where substantial intercept deviations were observed.

In TEST2, calibration intercepts shifted modestly in the negative direction for several models, indicating mild systematic overprediction consistent with lower event prevalence in the temporal cohort. Although calibration slopes remained close to unity for LR and ensemble models, greater variability was observed overall in the temporal validation cohort, particularly for the DT and NB models. Collectively, calibration was more stable in TEST1 than in TEST2.

### Threshold-based Performance

Using TRAIN-derived thresholds, external validation demonstrated interpretable sensitivity–specificity trade-offs (Supplementary Table A9). For in-hospital mortality, sensitivity in TEST1 ranged from 0.786 to 0.885 for most models, with specificity between 0.868 and 0.936. In TEST2, sensitivity decreased to 0.523–0.723 while specificity remained relatively preserved (0.869–0.959), indicating modest temporal attenuation primarily affecting case detection rather than false-positive control.

For 30-day mortality, a similar pattern was observed. In TEST1, sensitivity ranged from 0.788 to 0.815 and specificity from 0.883 to 0.901 for LR and ensemble models. In TEST2, sensitivity declined to 0.491–0.628, whereas specificity remained high (0.876–0.933). BoostedDT achieved the highest MCC for short-term endpoints in TEST1 (MCC 0.540 for in-hospital; 0.526 for 30-day), whereas BaggedDT demonstrated comparatively greater stability in TEST2.

For 1-year mortality, sensitivity was lower overall (0.669–0.692 in TEST1 and 0.510–0.569 in TEST2), reflecting greater outcome uncertainty. Ensemble models achieved modest improvements in balanced classification, while LR demonstrated more consistent performance across cohorts. Across endpoints, temporal attenuation primarily affected sensitivity rather than specificity.

### Decision Curve Analysis

**Fig. 4:**
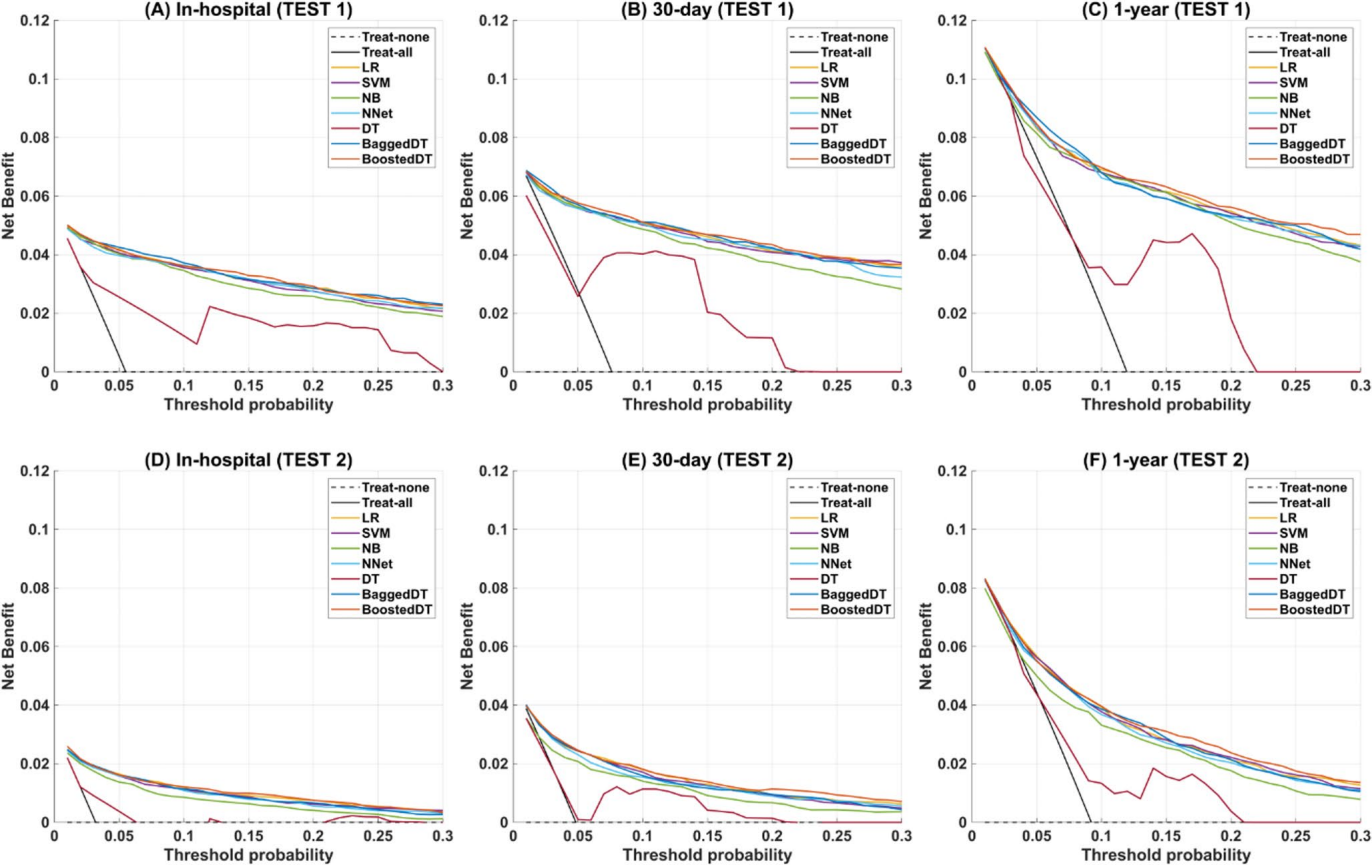
Decision curve analysis showing net benefit across threshold probabilities (0–30%) for in-hospital, 30-day, and 1-year mortality in TEST1 (**A**-**C**) and TEST2 (**D**-**F**). Treat-all and treat-none strategies are shown

Decision curve analysis demonstrated positive net benefit for most multivariable models across clinically relevant threshold probabilities (0–30%) in both hospital-level (TEST1) and temporal (TEST2) validation cohorts (Fig. 4).

For in-hospital and 30-day mortality, LR and ensemble tree-based models consistently provided greater net benefit than treat-all and treat-none strategies across low-to-intermediate thresholds (approximately 5–25%). Net benefit attenuated in TEST2, consistent with lower event prevalence, but relative model ordering was largely preserved.

For 1-year mortality, net benefit remained positive across low-to-intermediate thresholds in both validation cohorts, although absolute net benefit values were smaller than for short-term endpoints. Model curves were more closely clustered, indicating broadly comparable clinical utility across modeling approaches.

Overall, decision curve analysis supports incremental clinical benefit of the models for mortality risk stratification within threshold ranges relevant to triage and early management decisions.

### Cross-Model Stable Predictors Across Mortality Time Horizons

**Fig. 5:**
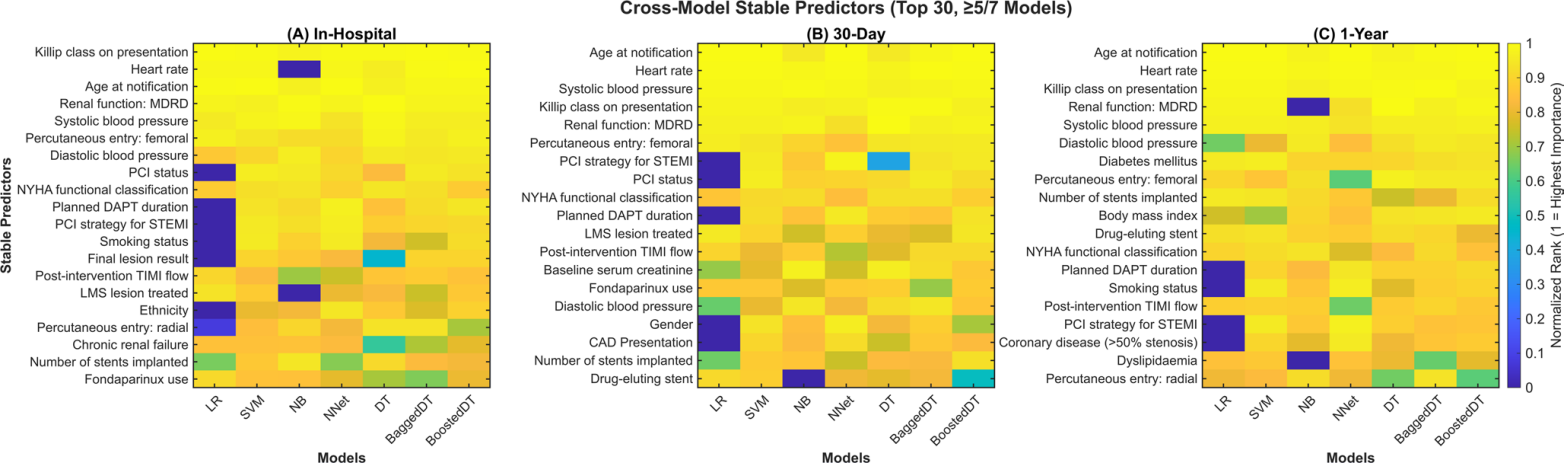
Cross-model stable predictors for (**A**) in-hospital, (**B**) 30-day, and (C) 1-year mortality. Heatmaps display normalized block-level permutation importance ranks (0–1; higher = greater importance) for predictors ranked within the top 30 in ≥ 5 of 7 models. Rows represent predictor blocks; columns represent models

Cross-model stability analysis identified a coherent, clinically interpretable set of predictor blocks consistently ranked highly across ML models and endpoints (Fig. 5; Supplementary Table A10). Across all three time horizons, the strongest consensus was observed for variables reflecting acute illness severity, haemodynamic compromise, and baseline physiological vulnerability.

Age at notification showed the strongest consensus for longer-term risk, ranking first for both 30-day and 1-year mortality (median rank = 1 across models), suggesting a dominant influence on intermediate and long-term outcomes. NYHA functional classification also demonstrated stability across all endpoints (7/7 models), reinforcing the importance of heart failure burden.

Procedural complexity and reperfusion success contributed consistent predictive value. Number of stents, left main stem treatment, and post-intervention TIMI flow were stable in ≥ 6/7 models for short-term endpoints. These likely reflect anatomical disease burden and procedural effectiveness.

Treatment-related variables, including PCI strategy for STEMI and planned dual antiplatelet therapy (DAPT) duration, also demonstrated cross-model stability. These variables likely capture integrated clinical risk assessment at the time of intervention, as treatment strategies are typically tailored to underlying disease severity and physician-perceived risk rather than representing causal treatment effects alone. Their repeated selection across diverse modelling approaches suggests real-world decision-making contains prognostic signal beyond isolated physiological measurements.

Temporal patterns differed modestly across endpoints. In-hospital and 30-day mortality were dominated by acute severity and haemodynamic markers (e.g., Killip class, heart rate, blood pressure), whereas 1-year mortality demonstrated greater prominence of chronic cardiometabolic factors, including diabetes and stent burden. Twelve predictor blocks met the stability criterion across all three endpoints (Table 3), being ranked within the top 30 in ≥ 5 of 7 models for each outcome.

**Table 3:** Cross-outcome stable predictor blocks identified by cross-model block-level permutation importance

Predictor	In-hospital	30-Day	1-Year	Stable in *n* outcomes
1. Diastolic blood pressure	✔	✔	✔	3
2. Systolic blood pressure	✔	✔	✔	3
3. Percutaneous entry: femoral	✔	✔	✔	3
4. Heart rate	✔	✔	✔	3
5. Killip class on presentation	✔	✔	✔	3
6. Renal function: MDRD	✔	✔	✔	3
7. Number of stents implanted	✔	✔	✔	3
8. NYHA functional classification	✔	✔	✔	3
9. PCI strategy for STEMI	✔	✔	✔	3
10. Planned DAPT duration	✔	✔	✔	3
11. Age at notification	✔	✔	✔	3
12. Post-intervention TIMI flow	✔	✔	✔	3
13. Fondaparinux use	✔	✔		2
14. PCI status	✔	✔		2
15. Percutaneous entry: radial	✔		✔	2
16. Smoking status	✔		✔	2
17. Drug-eluting stent		✔	✔	2
18. LMS lesion treated	✔	✔		2
19. CAD Presentation		✔		1
20. Body mass index			✔	1
21. Diabetes mellitus			✔	1
22. Dyslipidaemia			✔	1
23. Baseline serum creatinine		✔		1
24. Chronic renal failure	✔			1

Check marks indicate predictor blocks ranked within the top 30 by block permutation ROC-AUC decrease in ≥ 5 of 7 models for each endpoint. “Stable in n outcomes” denotes the number of endpoints (1–3) meeting this criterion

## Discussion

In this nationwide multicentre analysis of 29,521 ACS patients undergoing PCI, seven ML models demonstrated strong discrimination for in-hospital mortality and moderate discrimination for 30-day and 1-year mortality. Performance remained clinically meaningful under both geographical and prospective temporal validation. Cross-model stability analysis identified a clinically interpretable set of predictors reflecting acute illness severity, haemodynamic compromise, and baseline physiological vulnerability.

Discrimination was highest for in-hospital mortality and declined progressively for longer-term outcomes. This temporal gradient is biologically plausible. Early mortality is largely driven by acute physiological instability and procedural factors, whereas longer-term mortality reflects evolving comorbidity burden, adherence patterns, and healthcare system influences that are not fully captured at baseline. The attenuation in discrimination over time therefore likely reflects increasing outcome complexity rather than model inadequacy.

No single algorithm consistently dominated across endpoints and validation cohorts. Although ensemble methods achieved marginally higher ROC-AUC values in selected settings, the differences were small and not consistently clinically meaningful. This finding underscores that robustness, stability, and transportability across datasets may be more important than peak apparent performance within a single cohort. The weaker performance of the single decision tree model across time horizons further supports this interpretation. Its lower performance demonstrates the limitations of simple, un-ensembled rule-based partitioning when applied to complex, sparse, and imbalanced registry data, while also highlighting the added value of ensemble and nonlinear frameworks in capturing higher-order clinical interactions.

Calibration performance underscored the importance of external evaluation. In temporal validation, modest negative intercept shifts indicated mild overprediction consistent with lower contemporary event rates. Logistic recalibration based on the development cohort shifted calibration intercepts toward zero and slopes toward unity in validation datasets, supporting model updating without full retraining. These findings highlight that strong discrimination does not ensure accurate absolute risk estimation, particularly when clinical decisions are guided by predefined thresholds. Reporting Brier scores alongside discrimination and calibration metrics provides a more complete assessment of predictive performance. The attenuation observed in TEST2 may reflect changes in PCI practice, pharmacotherapy, and case mix over time, emphasizing the need for ongoing monitoring and periodic recalibration in real-world deployment.

When benchmarked against published post-PCI mortality models (Table 4), the present models demonstrated competitive discrimination under more rigorous validation settings. For in-hospital mortality, ROC-AUC in hospital-level external validation (0.934–0.943) was within the upper range of previously reported values (0.79–0.96), most of which were derived using internal validation. Similarly, 30-day mortality performance in TEST1 (0.917–0.923) and temporal validation (0.834–0.838) remained comparable to or exceeded several internally validated ML studies. For 1-year mortality, discrimination in external validation (0.852–0.859 in TEST1; 0.798–0.801 in TEST2) aligned with published registry-based models despite differences in case mix and event rates. Importantly, many prior studies relied on random data splits, whereas the current study incorporated hospital-level and prospective temporal validation, providing a more stringent assessment of transportability.

**Table 4:** Benchmarking of proposed models against published post-PCI mortality studies

A. In-hospital mortality						
Study	**Country**	**ML Method**	**Validation Set (n)**	**Mortality (%)**	**ROC-AUC**	**Validation Level**
Peterson et al. [10]	USA	LR	285,440	1.17	0.924	Internal Temporal
Galimzhanov et al. [11]	USA	XGBoost	331,990	2.2	0.86	Internal (Random Split)
Sritharan et al. [12]	Australia	Elastic Net	373	6.9	0.79	Internal (Random Split)
Soleimani et al. [13]	Iran	RF	2,227	1.74	0.924	Internal (Random Split)
Niimi et al. [14]	Japan	XGBoost	5,740	2.3	0.955	Internal (Random Split)
Soong et al. [15]	Singapore	XGBoost	346	8.5	0.83	Internal (Random Split)
Current study (TEST1)	Malaysia	LR/BaggedDT/BoostedDT**	4,246	5.51	0.934-0.943	External (Geographical – Hospital Split)
Current study (TEST2)	Malaysia	LR/BaggedDT/BoostedDT**	8,289	3.18	0.874-0.884	External (Prospective Multicenter)
B. 30-day mortality						
Study	**Country**	**ML Method**	**Validation Set (n)**	**Mortality (%)**	**ROC-AUC**	**Validation Level **
Hizoh et al. [5]	Hungary	LR	505	8.1	0.872	Internal Temporal
Burrello et al. [16]	Italy	RF	1,701	0.35	0.816	External (Independent Cohort)
Khan Chowdhury et al. [17]	Australia	RF	9,306	2.1	0.943	Internal (Random Split)
Current study (TEST1)	Malaysia	LR/BaggedDT/BoostedDT**	3,832	7.62	0.917-0.923	External (Geographical – Hospital Split)
Current study (TEST2)	Malaysia	LR/BaggedDT/BoostedDT**	8,282	4.84	0.834-0.838	External (Prospective Multicenter)
C. 1-Year Mortality						
Study	**Country**	**ML Method**	**Validation Set (n)**	**Mortality (%)**	**ROC-AUC**	**Validation Level **
Maluenda et al. [18]	USA	LR	973	10.3	0.836	Internal Temporal
Burrello et al. [16]	Italy	RF	1701 (external)	1.29%	0.691	External (Independent Cohort)
Hosseini et al. [6]	Iran	RF	2,722	3.7	0.866	Internal (Random Split)
Current study (TEST1)	Malaysia	LR/BaggedDT/BoostedDT**	3,688	11.96	0.852-0.859	External (Geographical – Hospital Split)
Current study (TEST2)	Malaysia	LR/BaggedDT/BoostedDT**	8,281	9.21	0.798-0.801	External (Prospective Multicenter)

**Models demonstrated similar discrimination (ΔAUC < 0.05)

Despite algorithmic heterogeneity, a consistent set of predictors emerged across models and mortality endpoints. Stable predictors included markers of haemodynamic compromise (e.g., Killip class), age, renal function, heart rate, and systolic blood pressure. Their recurrent importance across linear, kernel-based, probabilistic, neural network, and ensemble tree-based architectures supports both statistical robustness and clinical validity. Pathophysiologically, these variables capture the interplay between baseline physiological reserve, acute myocardial injury severity, and systemic comorbidity burden. Acute instability markers were most influential for in-hospital mortality, whereas longer-term outcomes showed relatively greater contribution from chronic cardiometabolic factors, suggesting a transition from instability-driven to vulnerability-driven risk.

Decision curve analysis further demonstrated positive net benefit across clinically relevant threshold probabilities in both validation cohorts. Across thresholds of approximately 5–25%, multivariable models consistently provided greater net benefit than default treat-all or treat-none strategies, indicating improved identification of high-risk patients without disproportionate false positives. Although net benefit attenuated in temporal validation, consistent with lower event prevalence, model ranking and relative clinical utility were largely preserved. These findings suggest potential value in supporting intensified monitoring, discharge optimization, and secondary prevention strategies. However, integration into clinical workflows would require prospective impact assessment.

This study contributes to the evolving literature on ML-based cardiovascular risk prediction by combining large-scale multicentre data, rigorous external validation, calibration assessment, benchmarking against prior studies, and cross-model interpretability within a multiethnic Asian PCI population. Nonetheless, several limitations warrant consideration. As a registry-based analysis, residual confounding and unmeasured variables cannot be excluded. Rare-event prediction remains challenging despite methodological safeguards, and longer-term mortality may be influenced by post-discharge factors not captured at baseline. Incomplete follow-up or delayed registry updates may introduce residual outcome misclassification despite linkage with national death records. Finally, although geographical and temporal validation were performed, independent international validation would further strengthen generalisability.

Overall, these findings support the translational potential of ML-based mortality prediction following PCI while emphasising the importance of external validation, calibration monitoring, and ongoing performance assessment for real-world implementation.

## Conclusions

In this large, multiethnic Southeast Asian PCI cohort, seven ML models demonstrated strong discrimination for in-hospital mortality and moderate performance for 30-day and 1-year mortality, with competitive results compared to previously published studies. Performance remained clinically meaningful in both geographical and temporal external validation cohorts. Logistic recalibration derived from the development cohort improved calibration and supported model transportability. Cross-model stability analysis identified consistent, clinically plausible predictors across endpoints. Together with positive net benefit demonstrated in decision curve analysis, these findings support the potential role of ML-based risk stratification in patients undergoing PCI for acute coronary syndromes within diverse Asian healthcare settings. Implementation should incorporate external validation, calibration monitoring, and periodic performance reassessment to ensure sustained reliability in evolving clinical environments.

## Supplementary Information

Below is the link to the electronic supplementary material.

Supplementary Material 1 (DOCX 86.5 KB)

## Data Availability

NCVD-PCI registry data are managed by the NHAM in collaboration with the Ministry of Health Malaysia and are not publicly available due to privacy and ethical restrictions. Access requires approval from the registry authorities upon reasonable request.
