# Supplementary material for: Predicting Mortality After Percutaneous Coronary Intervention in a Multiethnic Southeast Asian Population: Insights From Machine Learning
Source: J Cardiovasc Transl Res. 2026 Jul 6;19(1):84. doi: 10.1007/s12265-026-10812-5 (PMC13337946; doi:10.1007/s12265-026-10812-5)
Supplement: Supplementary file 1 — Supplementary Material 1 (DOCX 86.5 KB) [file 12265_2026_10812_MOESM1_ESM.docx]

**Supplementary Materials**

**Table A1. Predictor definitions, transformations, and data handling**

|  | **Variable name** | **Description** | **Data type** | **Values / Acceptable range** |
| --- | --- | --- | --- | --- |
| **Section 1: Demographics** | | | | |
| 1 | ptsex | Gender | Categorical (binary) | Male, Female |
| 2 | ptrace | Ethnicity | Categorical (nominal) | Malay, Chinese, Indian, Others (all remaining registry categories grouped) |
| 3 | ptageatnotification | Age at notification | Continuous | Years |
| **Section 2: Status before event** | | | | |
| 4 | smokingstatus | Smoking status | Categorical (nominal) | Current (any tobacco use within last 30 days), former (quit > 30 days), never |
| 5 | cdys | Dyslipidaemia | Binary | True/False |
| 6 | chpt | Hypertension | Binary | True/False |
| 7 | cdm | Diabetes mellitus | Binary | True/False |
| 8 | cdmtoha | Diabetes treatment: Oral hypoglycaemic agents (OHA) | Binary | True/False |
| 9 | cdmtinsulin | Diabetes treatment: Insulin | Binary | True/False |
| 10 | cdmtnonpharma | Diabetes treatment: Non-pharmacological (diet/lifestyle) | Binary | True/False |
| 11 | cpremcvd | Family history of premature cardiovascular disease | Binary | True/False |
| 12 | cmi | History of myocardial infarction | Binary | True/False |
| 13 | cdcad | Documented coronary artery disease | Binary | True/False |
| 14 | canginapast2wk | New-onset angina within past 2 weeks | Binary | True/False |
| 15 | cheartfail | History of heart failure | Binary | True/False |
| 16 | ccerebrovascular | Cerebrovascular disease | Binary | True/False |
| 17 | cpvascular | Peripheral vascular disease | Binary | True/False |
| 18 | crenalfail | Chronic renal failure | Binary | True/False |
| **Section 3: Baseline investigation** | | | | |
| 19 | bmi | Body mass index | Continuous | 14–50 kg/m² |
| 20 | heartrate | Heart Rate | Continuous | 25-200 beats/min |
| 21 | bpsys | Systolic blood pressure | Continuous | 60–230 mmHg |
| 22 | bpdias | Diastolic blood pressure | Continuous | 10–120 mmHg |
| 23 | creat | Baseline serum creatinine | Continuous | 44–2000 µmol/L |
| 24 | ecgsinus | Baseline ECG: Sinus rhythm | Binary | True/False |
| 25 | ecgatrial | Baseline ECG: Atrial fibrillation | Binary | True/False |
| 26 | ecgavb | Baseline ECG: Second- or third-degree AV block | Binary | True/False |
| 27 | ecglbbb | Baseline ECG: Left bundle branch block (LBBB) | Binary | True/False |
| 28 | ecgrbbb | Baseline ECG: Right bundle branch block (RBBB) | Binary | True/False |
| 29 | mdrd | Estimated glomerular filtration rate (GFR): MDRD | Continuous | 1–200 mL/min/1.73 m² |
| 30 | cg | Estimated glomerular filtration rate (GFR): Cockcroft-Gault | Continuous | 1-200mL/min |
| **Section 4: Previous Intervention** | | | | |
| 31 | previouspci | Previous percutaneous coronary intervention | Binary | True/False |
| 32 | previouscabg | Previous coronary artery bypass graft surgery | Binary | True/False |
| **Section 5: Cardiac status at PCI procedure** | | | | |
| 33 | nyha | NYHA functional classification | Ordinal | nyha I, nyha II, nyha III, nyha IV |
| 34 | killipclass | Killip class on presentation | Ordinal | killip I, killip II, killip III, killip IV |
| 35 | acsyes | CAD Presentation | Categorical (nominal) | STEMI, NSTEMI, UA |
| 36 | stemi | ECG STEMI location | Categorical (binary) | Anterior, non-anterior |
| 37 | acsanginatype | Angina type | Categorical (nominal) | Typical, atypical, none |
| 38 | ccs | Canadian Cardiovascular Society (CCS) angina class | Ordinal | ccs 0(asymptomatic), ccs 1, ccs 2, ccs 3, ccs 4 |
| **Section 6: Cath Lab Visit** | | | | |
| 39 | pcistatus | PCI status | Categorical (binary) | NSTEMI/UA, STEMI |
| 40 | pcistemi | PCI strategy for STEMI | Categorical (nominal) | Rescue, Primary, Facilitated, Delayed |
| 41 | mthrombolytics | Thrombolytic therapy prior to PCI | Binary | True/False |
| 42 | mblockade | GP IIb/IIIa inhibitor use | Binary | True/False |
| 43 | mheparin | Unfractionated heparin use | Binary | True/False |
| 44 | mlmwh | Low-molecular-weight heparin use | Binary | True/False |
| 45 | mticlopidine | Ticlopidine use | Binary | True/False |
| 46 | mbivalirudin | Bivalirudin use | Binary | True/False |
| 47 | maspirin | Aspirin use | Binary | True/False |
| 48 | mclopidogrel | Clopidogrel use | Binary | True/False |
| 49 | mfondaparinox | Fondaparinux use | Binary | True/False |
| 50 | plandurclopticlop | Planned duration of clopidogrel / ticlopidine | Ordinal | 1 month, 3 months, 6 months, 12 months, >12 months |
| 51 | brachial | Percutaneous entry: brachial | Binary | True/False |
| 52 | radial | Percutaneous entry: radial | Binary | True/False |
| 53 | femoral | Percutaneous entry: femoral | Binary | True/False |
| 54 | ulnar | Percutaneous entry: ulnar | Binary | True/False |
| 55 | extentcd_all_group | Coronary disease with >50% stenosis | Categorical (nominal) | Graft, LMS, single, multiple |
| **Section 7: PCI Procedure Details** | | | | |
| ***Lesion & vessel Characteristics*** | | | | |
| 56 | lesionnumber | Total number of lesions treated | Integer | Count |
| 57 | treated_LMS_lesion | LMS lesion treated | Binary | True/False |
| 58 | treated_LAD_lesion | LAD lesion treated | Binary | True/False |
| 59 | treated_LCx_lesion | LCx lesion treated | Binary | True/False |
| 60 | treated_RCA_lesion | RCA lesion treated | Binary | True/False |
| 61 | treated_Graft_lesion | Graft lesion treated | Binary | True/False |
| 62 | n_treated_vessels | Number of vessels treated | Integer | Count |
| 63 | any_multivessel_PCI | Multivessel PCI | Binary | True/False |
| ***Lesion type & complexity*** | | | | |
| 64 | is_denovo | De novo lesion | Binary | True/False |
| 65 | is_instent_restenosis | In-stent Restenosis | Binary | True/False |
| 66 | is_stent_thrombosis | Stent thrombosis | Binary | True/False |
| 67 | is_restenosis_no_prior_stent | Restenosis without prior stent | Binary | True/False |
| 68 | lesiontype_A | ACC/AHA lesion type A | Binary | True/False |
| 69 | lesiontype_B1 | ACC/AHA lesion type B1 | Binary | True/False |
| 70 | lesiontype_B2 | ACC/AHA lesion type B2 | Binary | True/False |
| 71 | lesiontype_C | ACC/AHA lesion type C | Binary | True/False |
| **Lesion location & morphology** | | | | |
| 72 | locationingraft_ostial | Graft lesion: ostial | Binary | True/False |
| 73 | locationingraft_body | Graft lesion: body | Binary | True/False |
| 74 | locationingraft_anastomosis | Graft lesion: anastomosis | Binary | True/False |
| 75 | locationingraft_native | Graft lesion: native vessel | Binary | True/False |
| 76 | lesionostial | Ostial lesion | Binary | True/False |
| 77 | lesionctolarger | Chronic total occlusion >3 months | Binary | True/False |
| 78 | lesionthrombus | Thrombus-containing lesion | Binary | True/False |
| 79 | lesionna | Lesion not applicable (Lesions for which specific morphological descriptors i.e. bifurcation, thrombus, chronic total occlusion, were not applicable) | Binary | True/False |
| 80 | lesionbifurcation | Bifurcation lesion | Binary | True/False |
| ***Angiographic severity & outcome*** | | | | |
| 81 | prestenosis | Pre-intervention stenosis | Continuous | 0-100% |
| 82 | timiflowpre | Pre-intervention TIMI flow | Ordinal | timi-0, timi-1, timi-2, timi-3 |
| 83 | poststenosis | Post-intervention stenosis | Continuous | 0-100% |
| 84 | timiflowpost | Post-intervention TIMI flow | Ordinal | timi-0, timi-1, timi-2, timi-3 |
| 85 | sum_lesion_length | Total lesion length per patient | Continuous | mm (no fixed upper bound; quality control applied at per-lesion level) |
| 86 | max_lesion_length | Maximum lesion length | Continuous | 1-150 mm |
| 87 | num_longlesion | Number of long lesions (>20 mm) | Integer | Count |
| 88 | dissection | Coronary dissection | Binary | True/False |
| 89 | perforation | Coronary perforation | Binary | True/False |
| 90 | noreflow | No-reflow phenomenon | Binary | True/False |
| 91 | lesionresult | Final lesion result | Categorical (binary) | Successful, unsuccessful |
| 92 | directstenting | Direct stenting | Binary | True/False |
| ***Stent Characteristics*** | | | | |
| 93 | total_stent_length | Total stent length per patient | Continuous | mm (no fixed upper bound; derived from individual stent lengths) |
| 94 | max_stent_length | Maximum stent length | Continuous | mm (no fixed upper bound; constrained by individual stent specifications) |
| 95 | n_stents | Number of stents implanted | Integer | Count |
| 96 | min_stent_diameter | Minimum stent diameter | Continuous | 2-6 mm |
| 97 | stent_DES | Drug-eluting stent | Binary | True/False |
| 98 | stent_BMS | Bare-metal stent | Binary | True/False |
| 99 | stent_BioAbsorb | Bioabsorbable stent | Binary | True/False |
| 100 | stent_Antibody | Antibody-coated stent | Binary | True/False |
| 101 | stent_Others | Other stent types | Binary | True/False |
| 102 | stent_DEB | Drug-eluting Balloon | Binary | True/False |
| 103 | stent_Bifurcated | Bifurcated stent | Binary | True/False |
| 104 | stent_Covered | Covered stent | Binary | True/False |
| 105 | stent_Combo | Combination stent | Binary | True/False |

**Table A2. Hospital-level allocation for model development and external validation**

| 1. **Hospital-based split (2007–2018)** | | |
| --- | --- | --- |
| **Hospital** | **Patients** | **Assigned set** |
| 1. Institut Jantung Negara, Kuala Lumpur | 4,293 | TRAIN  (n=16,986) |
| 1. Serdang Hospital, Selangor | 2,406 |  |
| 1. University Malaya Medical Centre, Kuala Lumpur | 1,989 |  |
| 1. Sultanah Bahiyah Hospital, Kedah | 1,825 |  |
| 1. Queen Elizabeth II Hospital, Sabah | 1,576 |  |
| 1. Sultanah Aminah Hospital, Johor | 1,386 |  |
| 1. Raja Permaisuri Bainun Hospital, Perak | 1,173 |  |
| 1. Tengku Ampuan Afzan Hospital, Pahang | 1,077 |  |
| 1. UKM Medical Centre, Kuala Lumpur | 335 |  |
| 1. Subang Jaya Medical Centre, Selangor | 280 |  |
| 1. UiTM Medical Centre, Selangor | 226 |  |
| 1. Sultanah Nur Zahirah Hospital, Terengganu | 228 |  |
| 1. Pantai Hospital Ipoh, Perak | 109 |  |
| 1. KPJ Tawakkal Specialist Hospital, Kuala Lumpur | 31 |  |
| 1. KPJ Selangor Specialist Hospital, Selangor | 22 |  |
| 1. KPJ Klang Specialist Hospital, Selangor | 22 |  |
| 1. Cardiac Vascular Sentral, Kuala Lumpur | 5 |  |
| 1. Queen Elizabeth Hospital, Sabah | 3 |  |
| 1. Sarawak General Hospital, Sarawak | 2,093 | TEST1 (external)  n = 4,246 |
| 1. Penang Hospital, Penang | 1,694 |  |
| 1. Raja Perempuan Zainab II Hospital, Kelantan | 376 |  |
| 1. Oriental Melaka Straits Medical Centre, Malacca | 66 |  |
| 1. KPJ Penang Specialist Hospital, Penang | 17 |  |
| **Total** | 21,232 | — |
| 1. **Temporal split (2019-2020)** | | |
| **Hospital** | Patients | Assigned set |
| 1. Institut Jantung Negara, Kuala Lumpur | 556 | TEST2  (temporal)  (n = 8,289) |
| 1. Serdang Hospital, Selangor | 1456 |  |
| 1. University Malaya Medical Centre, Kuala Lumpur | 534 |  |
| 1. Sultanah Bahiyah Hospital, Kedah | 674 |  |
| 1. Queen Elizabeth II Hospital, Sabah | 562 |  |
| 1. Sultanah Aminah Hospital, Johor | 347 |  |
| 1. Raja Permaisuri Bainun Hospital, Perak | 653 |  |
| 1. Tengku Ampuan Afzan Hospital, Pahang | 443 |  |
| 1. UKM Medical Centre, Kuala Lumpur | 227 |  |
| 1. Subang Jaya Medical Centre, Selangor | 24 |  |
| 1. UiTM Medical Centre, Selangor | 59 |  |
| 1. Sultanah Nur Zahirah Hospital, Terengganu | 53 |  |
| 1. KPJ Tawakkal Specialist Hospital, Kuala Lumpur | 6 |  |
| 1. KPJ Penang Specialist Hospital, Penang | 130 |  |
| 1. Cardiac Vascular Sentral, Kuala Lumpur | 304 |  |
| 1. Penang Hospital, Penang | 552 |  |
| 1. Raja Perempuan Zainab Ii Hospital, Kelantan | 415 |  |
| 1. Sarawak General Hospital, Sarawak | 950 |  |
| 1. Oriental Melaka Straits Medical Centre, Malacca | 46 |  |
| 1. KPJ Klang Specialist Hospital, Selangor | 88 |  |
| 1. KPJ Seremban Specialist Hospital | 73 |  |
| 1. Gleneagles Hospital Medini, Johor | 58 |  |
| 1. Pantai Hospital Penang, Penang | 48 |  |
| 1. Gleneagles Hospital Kota Kinabalu | 26 |  |
| 1. Gleneagles Hospital Penang, Penang | 5 |  |
| **Total** | 8,289 |  |

**Table A3. Hyperparameter search space and optimal configurations for all models. Hyperparameters were optimized using grid search within nested cross-validation on the training cohort. The total number of configurations evaluated per model is reported. Log-spaced ranges are expressed on the base-10 scale. Optimal values are shown separately for in-hospital, 30-day, and 1-year mortality endpoints.**

| **Model** | **Total Configuration Evaluated** | **Hyperparameter** | **Search Range** | **Best Value (In-hospital)** | **Best Value (30-day)** | **Best Value (1-year)** |
| --- | --- | --- | --- | --- | --- | --- |
| LR | 60 | λ | 10⁻⁶ to 10¹ (60 values, log-spaced) | 0.0036251 | 0.0047639 | 0.0027585 |
| SVM | 52 | KernelFunction | linear, RBF | linear | linear | linear |
|  |  | C | linear: 10⁻³ to 10³ (10 values, log-spaced)  rbf: 10⁻³ to 10³ (6 values, log-spaced) | 0.001 | 0.001 | 0.001 |
|  |  | KernelScale | [auto, 10⁻²–10² (6 values, log-spaced)] (RBF only) | - | - | - |
| NB | 120 | KTop | [10, 20, 30, 40, 50, 60, 80, 100, 120, all] | 60 | 80 | 60 |
|  |  | Kernel | Normal | normal | normal | normal |
|  |  | Mode | mixed, kernel | kernel | kernel | kernel |
|  |  | PriorType | empirical, uniform | empirical | empirical | empirical |
|  |  | Width | [0.2, 0.5, 1.0] | 0.5 | 1 | 1 |
| NeuralN | 36 | Activations | relu, tanh | relu | relu | relu |
|  |  | Iterations | [800, 1500] | 1500 | 800 | 800 |
|  |  | Lambda | [10⁻⁴, 10⁻³, 10⁻²] | 0.01 | 0.01 | 0.01 |
|  |  | LayerSizes | 1 hidden layer (20 neurons),  1 hidden layer (50 neurons),  2 hidden layers (50–20 neurons) | 50 | 20 | 20 |
|  |  | InitialStepSize | auto |  |  |  |
| DT | 32 | MaxNumSplits | [10, 30, 100, 300] | 300 | 100 | 300 |
|  |  | MinLeafSize | [5, 10, 20, 50] | 10 | 5 | 20 |
|  |  | SplitCriterion | gdi, deviance | deviance | deviance | deviance |
| BaggedDT | 54 | MaxNumSplits | [10, 30, 60] | 60 | 60 | 60 |
|  |  | MinLeafSize | [5, 10, 20] | 20 | 20 | 20 |
|  |  | NumLearningCycles | [100, 300, 600] | 600 | 300 | 600 |
|  |  | NumVariablesToSample | all, √p | sqrt | sqrt | sqrt |
| BoostedDT | 192 | Method | LogitBoost, GentleBoost, AdaBoostM1, RUSBoost | LogitBoost | LogitBoost | LogitBoost |
|  |  | LearnRate | [0.05, 0.1] | 0.05 | 0.1 | 0.1 |
|  |  | MaxNumSplits | [1, 3, 10, 30] | 30 | 3 | 3 |
|  |  | MinLeafSize | [5, 10] | 5 | 10 | 10 |
|  |  | NumLearningCycles | [100, 300, 600] | 300 | 600 | 600 |

**Table A4. Software environment and reproducibility settings**

| **Component** | **Description** |
| --- | --- |
| Programming language | MATLAB |
| MATLAB version | R2024b |
| Toolboxes | Statistics and Machine Learning Toolbox |
| Operating system | Linux |
| Random Seed | CV partitioning:   - rng(20260108) - tuning CV - rng(20260109) - evaluation CV   Block permutation importance:   - deterministic Threefry streams with seeds 20260225 |
| Cross-validation | Stratified K-fold CV (K = 5) on TRAIN set |
| Missing data handling | Continuous predictors imputed using **median** (TRAIN only). Categorical predictors imputed using **Missing** category (TRAIN-defined levels; unseen levels mapped to Missing). |
| Class imbalance handling | Sample weighting (inverse-frequency class weights; normalized to mean 1) applied to TRAIN only |
| Threshold selection | TRAIN only thresholding (e.g., Youden’s J; derived on TRAIN, then applied unchanged to TEST1/TEST2) |
| External validation | Hospital-based split (2007–2018) and temporal split (2019–2020) |

**Table A5. Baseline characteristics across TRAIN and external validation cohorts. Continuous variables are reported as median (IQR) and categorical variables as number (%). for continuous variables and the chi-square test for categorical variables. SMD indicates the absolute standardized mean difference versus TRAIN.**

| **Attributes** | **All cohorts**  **(2007–2020)** | **TRAIN**  **(2007–2018)** | **TEST1**  **(2007–2018)** | **TEST2**  **(2019–2020)** | **p-value** | **SMD (TEST1 vs TRAIN)** | **SMD (TEST2 vs TRAIN)** |
| --- | --- | --- | --- | --- | --- | --- | --- |
| Age (years) | 56.7 (48.9–64.4) | 56.7 (49.0–64.3) | 56.5 (48.7–64.5) | 56.7 (48.7–64.4) | 0.841 | 0.006 | 0.010 |
| Male, n (%) | 24873 (84.3%) | 14318 (84.3%) | 3586 (84.5%) | 6969 (84.1%) | 0.840 | 0.004 | 0.006 |
| Ethnicity, n (%) | Malay: 15155 (51.3%)  Chinese: 6014 (20.4%) Indian: 5658 (19.2%) Other Malaysian: 2690 (9.1%) | Malay: 8976 (52.9%)  Chinese: 3065 (18.0%) Indian: 3709 (21.8%) Other Malaysian: 1233 (7.3%) | Malay: 1908 (44.9%)  Chinese: 1260 (29.7%) Indian: 426 (10.0%) Other Malaysian: 651 (15.3%) | Malay: 4271 (51.5%)  Chinese: 1689 (20.4%) Indian: 1523 (18.4%) Other Malaysian: 806 (9.7%) | <0.001 | 0.327 | 0.089 |
| Body mass index (kg/m²) | 25.9 (23.5–28.9) | 25.9 (23.6–28.8) | 25.4 (22.9–28.2) | 26.1 (23.6–29.2) | <0.001 | 0.168 | 0.034 |
| Heart rate (beats/min) | 77.0 (66.0–89.0) | 77.0 (66.0–90.0) | 76.0 (66.0–88.0) | 75.0 (66.0–87.0) | <0.001 | 0.028 | 0.125 |
| Systolic blood pressure (mmHg) | 131.0 (114.0–150.0) | 130.0 (114.0–150.0) | 126.0 (111.0–144.0) | 134.0 (117.0–153.0) | <0.001 | 0.188 | 0.119 |
| Diastolic blood pressure (mmHg) | 77.0 (67.0–86.0) | 77.0 (67.0–87.0) | 73.0 (64.0–82.0) | 78.0 (69.0–87.0) | <0.001 | 0.266 | 0.068 |
| Baseline creatinine (µmol/L) | 90.0 (76.0–108.0) | 90.0 (76.0–110.0) | 87.0 (75.0–105.0) | 90.0 (76.8–106.0) | <0.001 | 0.086 | 0.002 |
| GFR (MDRD) | 78.7 (62.4–94.6) | 78.0 (60.9–94.5) | 80.4 (64.6–95.8) | 79.0 (63.7–94.1) | <0.001 | 0.088 | 0.030 |
| Smoking (current), n (%) | Current (Any Tobacco Use Within Last 30 Days): 11186 (45.0%) Former (Quit > 30 Days): 4902 (19.7%) Never: 8745 (35.2%) | Current (Any Tobacco Use Within Last 30 Days): 6394 (45.6%) Former (Quit > 30 Days): 2577 (18.4%) Never: 5037 (36.0%) | Current (Any Tobacco Use Within Last 30 Days): 1645 (43.2%) Former (Quit > 30 Days): 986 (25.9%) Never: 1178 (30.9%) | Current (Any Tobacco Use Within Last 30 Days): 3147 (44.9%) Former (Quit > 30 Days): 1339 (19.1%) Never: 2530 (36.1%) | <0.001 | 0.205 | 0.027 |
| Dyslipidaemia, n (%) | 13518 (51.7%) | 7454 (49.9%) | 2251 (61.1%) | 3813 (50.4%) | <0.001 | 0.226 | 0.009 |
| Hypertension, n (%) | 17228 (63.2%) | 9873 (63.0%) | 2557 (66.1%) | 4798 (62.1%) | <0.001 | 0.066 | 0.019 |
| Diabetes mellitus, n (%) | 11687 (43.2%) | 7042 (45.3%) | 1477 (38.3%) | 3168 (41.3%) | <0.001 | 0.143 | 0.079 |
| Family history premature CVD, n (%) | 3471 (14.3%) | 1853 (13.4%) | 651 (18.7%) | 967 (13.8%) | <0.001 | 0.143 | 0.010 |
| History of myocardial infarction, n (%) | 9054 (33.0%) | 5077 (32.6%) | 1633 (40.8%) | 2344 (29.7%) | <0.001 | 0.172 | 0.061 |
| Documented CAD, n (%) | 8089 (29.1%) | 4518 (28.6%) | 1129 (28.0%) | 2442 (30.7%) | <0.001 | 0.013 | 0.046 |
| New-onset angina (past 2 weeks), n (%) | 16582 (58.7%) | 9016 (56.2%) | 2354 (56.7%) | 5212 (64.7%) | <0.001 | 0.009 | 0.175 |
| History of heart failure, n (%) | 1016 (3.6%) | 596 (3.7%) | 152 (3.7%) | 268 (3.4%) | 0.397 | 0.003 | 0.019 |
| Cerebrovascular disease, n (%) | 638 (2.3%) | 409 (2.6%) | 81 (1.9%) | 148 (1.9%) | 0.001 | 0.041 | 0.047 |
| Peripheral vascular disease, n (%) | 147 (0.5%) | 95 (0.6%) | 23 (0.6%) | 29 (0.4%) | 0.062 | 0.006 | 0.034 |
| Chronic renal failure, n (%) | 1310 (4.7%) | 811 (5.1%) | 133 (3.2%) | 366 (4.6%) | <0.001 | 0.094 | 0.023 |
| Previous PCI, n (%) | 3073 (10.4%) | 1963 (11.6%) | 315 (7.4%) | 795 (9.6%) | <0.001 | 0.141 | 0.064 |
| Previous CABG, n (%) | 485 (1.6%) | 348 (2.0%) | 32 (0.8%) | 105 (1.3%) | <0.001 | 0.110 | 0.061 |
| Clinical presentation at PCI, n (%) | STEMI: 18273 (65.1%)  NSTEMI: 7202 (25.6%) UA: 2612 (9.3%) | STEMI: 10725 (67.3%)  NSTEMI: 3845 (24.1%) UA: 1358 (8.5%) | STEMI: 2651 (68.5%)  NSTEMI: 781 (20.2%) UA: 438 (11.3%) | STEMI: 4897 (59.1%)  NSTEMI: 2576 (31.1%) UA: 816 (9.8%) | <0.001 | 0.105 | 0.191 |

**Table A6. Discrimination performance on external validation (TEST1 and TEST2). ROC-AUC and PR-AUC are reported for (A) in-hospital, (B) 30-day, and (C) 1-year mortality. Values are estimates with 95% bootstrap confidence intervals.**

| **A. In-Hospital Mortality** | | | | |
| --- | --- | --- | --- | --- |
| **Model** | **ROC-AUC (TEST1)** | **ROC-AUC (TEST2)** | **PR-AUC (TEST1)** | **PR-AUC (TEST2)** |
| LR | 0.934 (0.916–0.950) | 0.874 (0.851–0.896) | 0.564 (0.491–0.626) | 0.318 (0.257–0.373) |
| SVM | 0.929 (0.911–0.948) | 0.870 (0.847–0.892) | 0.545 (0.471–0.615) | 0.311 (0.252–0.368) |
| NB | 0.927 (0.905–0.943) | 0.834 (0.807–0.861) | 0.483 (0.422–0.542) | 0.235 (0.186–0.282) |
| NNet | 0.919 (0.895–0.940) | 0.865 (0.842–0.887) | 0.552 (0.482–0.622) | 0.314 (0.258–0.370) |
| DT | 0.781 (0.745–0.815) | 0.681 (0.647–0.717) | 0.334 (0.278–0.388) | 0.154 (0.119–0.197) |
| BaggedDT | 0.943 (0.928–0.956) | 0.878 (0.856–0.898) | 0.560 (0.492–0.625) | 0.318 (0.259–0.378) |
| BoostedDT | 0.940 (0.924–0.954) | 0.884 (0.862–0.904) | 0.585 (0.514–0.648) | 0.301 (0.242–0.357) |
| **B. 30-Day Mortality** | | | | |
| **Model** | **ROC-AUC (TEST1)** | **ROC-AUC (TEST2)** | **PR-AUC (TEST1)** | **PR-AUC (TEST2)** |
| LR | 0.917 (0.896–0.933) | 0.834 (0.814–0.855) | 0.633 (0.573–0.690) | 0.311 (0.266–0.360) |
| SVM | 0.911 (0.892–0.930) | 0.832 (0.812–0.852) | 0.627 (0.570–0.686) | 0.293 (0.249–0.340) |
| NB | 0.903 (0.881–0.923) | 0.753 (0.725–0.784) | 0.585 (0.527–0.638) | 0.222 (0.184–0.264) |
| NNet | 0.902 (0.879–0.923) | 0.815 (0.792–0.837) | 0.608 (0.548–0.667) | 0.293 (0.247–0.341) |
| DT | 0.820 (0.785–0.854) | 0.739 (0.709–0.769) | 0.456 (0.390–0.520) | 0.188 (0.157–0.220) |
| BaggedDT | 0.923 (0.903–0.940) | 0.836 (0.814–0.856) | 0.633 (0.572–0.695) | 0.303 (0.254–0.351) |
| BoostedDT | 0.923 (0.904–0.940) | 0.838 (0.817–0.858) | 0.646 (0.585–0.702) | 0.320 (0.270–0.366) |
| **C. 1-Year Mortality** | | | | |
| **Model** | **ROC-AUC (TEST1)** | **ROC-AUC (TEST2)** | **PR-AUC (TEST1)** | **PR-AUC (TEST2)** |
| LR | 0.852 (0.831–0.871) | 0.801 (0.785–0.817) | 0.574 (0.524–0.617) | 0.361 (0.327–0.397) |
| SVM | 0.848 (0.827–0.868) | 0.799 (0.782–0.815) | 0.564 (0.514–0.610) | 0.351 (0.314–0.386) |
| NB | 0.833 (0.809–0.855) | 0.750 (0.728–0.769) | 0.530 (0.479–0.578) | 0.289 (0.258–0.320) |
| NNet | 0.843 (0.822–0.865) | 0.786 (0.768–0.805) | 0.558 (0.506–0.606) | 0.343 (0.309–0.375) |
| DT | 0.755 (0.725–0.784) | 0.708 (0.686–0.729) | 0.428 (0.381–0.473) | 0.261 (0.231–0.291) |
| BaggedDT | 0.854 (0.834–0.874) | 0.798 (0.781–0.814) | 0.565 (0.513–0.613) | 0.341 (0.304–0.375) |
| BoostedDT | 0.859 (0.840–0.880) | 0.799 (0.780–0.815) | 0.590 (0.541–0.640) | 0.356 (0.321–0.391) |

**Table A7. Pairwise DeLong comparisons of ROC-AUC between LR and alternative models in external validation cohorts.**

| **A. In-Hospital Mortality** | | | | | | |
| --- | --- | --- | --- | --- | --- | --- |
| **Split** | **Comparator** | **AUC (LR)** | **AUC (Comparator)** | **ΔAUC (LR − Comparator)** | **Z-statistic** | **p-value** |
| TEST1 | SVM | 0.934 | 0.929 | 0.005 | 2.74 | 0.006 |
|  | NB | 0.934 | 0.927 | 0.007 | 0.97 | 0.333 |
|  | NNet | 0.934 | 0.919 | 0.015 | 3.35 | <0.001 |
|  | DT | 0.934 | 0.781 | 0.153 | 8.99 | <0.001 |
|  | BaggedDT | 0.934 | 0.943 | -0.009 | -1.86 | 0.062 |
|  | BoostedDT | 0.934 | 0.94 | -0.007 | -1.47 | 0.142 |
| TEST2 | SVM | 0.874 | 0.87 | 0.003 | 1.63 | 0.104 |
|  | NB | 0.874 | 0.834 | 0.04 | 3.2 | 0.001 |
|  | NNet | 0.874 | 0.865 | 0.008 | 1.71 | 0.088 |
|  | DT | 0.874 | 0.681 | 0.192 | 10.39 | <0.001 |
|  | BaggedDT | 0.874 | 0.878 | -0.004 | -0.65 | 0.515 |
|  | BoostedDT | 0.874 | 0.884 | -0.01 | -1.68 | 0.094 |
| **B. 30-Day Mortality** | | | | | | |
| **Split** | **Comparator** | **AUC (LR)** | **AUC (Comparator)** | **ΔAUC (LR − Comparator)** | **Z-statistic** | **p-value** |
| TEST1 | SVM | 0.917 | 0.911 | 0.006 | 2.71 | 0.007 |
|  | NB | 0.917 | 0.903 | 0.014 | 1.53 | 0.126 |
|  | NNet | 0.917 | 0.902 | 0.015 | 3.07 | 0.002 |
|  | DT | 0.917 | 0.82 | 0.097 | 5.99 | <0.001 |
|  | BaggedDT | 0.917 | 0.923 | -0.006 | -1.22 | 0.221 |
|  | BoostedDT | 0.917 | 0.923 | -0.006 | -1.67 | 0.096 |
| TEST2 | SVM | 0.834 | 0.832 | 0.002 | 1 | 0.316 |
|  | NB | 0.834 | 0.753 | 0.081 | 5.57 | <0.001 |
|  | NNet | 0.834 | 0.815 | 0.019 | 2.99 | 0.003 |
|  | DT | 0.834 | 0.739 | 0.095 | 6.75 | <0.001 |
|  | BaggedDT | 0.834 | 0.836 | -0.002 | -0.24 | 0.811 |
|  | BoostedDT | 0.834 | 0.838 | -0.004 | -0.68 | 0.495 |
| **C. 1-Year Mortality** | | | | | | |
| **Split** | **Comparator** | **AUC (LR)** | **AUC (Comparator)** | **ΔAUC (LR − Comparator)** | **Z-statistic** | **p-value** |
| TEST1 | SVM | 0.852 | 0.848 | 0.003 | 2.48 | 0.013 |
|  | NB | 0.852 | 0.833 | 0.019 | 2.15 | 0.032 |
|  | NNet | 0.852 | 0.843 | 0.009 | 2.13 | 0.033 |
|  | DT | 0.852 | 0.755 | 0.097 | 7.32 | <0.001 |
|  | BaggedDT | 0.852 | 0.854 | -0.002 | -0.38 | 0.705 |
|  | BoostedDT | 0.852 | 0.859 | -0.008 | -1.71 | 0.087 |
| TEST2 | SVM | 0.801 | 0.799 | 0.002 | 1.46 | 0.144 |
|  | NB | 0.801 | 0.75 | 0.052 | 5.84 | <0.001 |
|  | NNet | 0.801 | 0.786 | 0.015 | 3.48 | <0.001 |
|  | DT | 0.801 | 0.708 | 0.093 | 9.04 | <0.001 |
|  | BaggedDT | 0.801 | 0.798 | 0.003 | 0.58 | 0.562 |
|  | BoostedDT | 0.801 | 0.799 | 0.003 | 0.58 | 0.562 |

**Table A8. Calibration performance on external validation (TEST1 and TEST2). Calibration is summarised by Brier score, calibration intercept, and calibration slope for (A) in-hospital, (B) 30-day, and (C) 1-year mortality. Values are estimates with 95% bootstrap confidence intervals.**

| **A. In-Hospital Mortality** | | | | | | | | |
| --- | --- | --- | --- | --- | --- | --- | --- | --- |
| **Model** | **Brier**  **(TEST1)** | **Brier**  **(TEST2)** | **Intercept**  **(TEST1)** | **Intercept**  **(TEST2)** | | **Slope**  **(TEST1)** | **Slope**  **(TEST2)** | |
| LR | 0.033 (0.029–0.038) | 0.026 (0.023–0.028) | -0.103 (-0.328–0.127) | -0.101 (-0.369–0.148) | | 0.944 (0.864–1.039) | 0.968 (0.887–1.057) | |
| SVM | 0.034 (0.030–0.038) | 0.026 (0.023–0.029) | -0.216 (-0.438–0.012) | -0.275 (-0.502–-0.035) | | 0.882 (0.805–0.979) | 0.906 (0.832–0.986) | |
| NB | 0.035 (0.031–0.039) | 0.027 (0.024–0.030) | -0.042 (-0.286–0.232) | -0.011 (-0.325–0.296) | | 0.987 (0.898–1.077) | 0.930 (0.838–1.026) | |
| NNet | 0.033 (0.029–0.038) | 0.026 (0.023–0.029) | -0.145 (-0.376–0.078) | -0.267 (-0.489–-0.035) | | 0.921 (0.836–1.020) | 0.937 (0.864–1.019) | |
| DT | 0.045 (0.041–0.050) | 0.031 (0.028–0.034) | 0.093 (-0.250–0.419) | -1.212 (-1.605–-0.867) | | 1.110 (0.962–1.268) | 0.768 (0.619–0.906) | |
| BaggedDT | 0.033 (0.029–0.037) | 0.026 (0.023–0.029) | -0.046 (-0.273–0.182) | 0.153 (-0.119–0.407) | | 1.024 (0.937–1.117) | 0.957 (0.879–1.041) | |
| BoostedDT | 0.032 (0.028–0.036) | 0.026 (0.023–0.029) | -0.122 (-0.355–0.099) | -0.232 (-0.477–0.026) | | 0.975 (0.895–1.072) | 0.940 (0.868–1.025) | |
| **B. 30-Day Mortality** | | | | | | | | |
| **Model** | **Brier**  **(TEST1)** | **Brier**  **(TEST2)** | **Intercept**  **(TEST1)** | **Intercept**  **(TEST2)** | | **Slope**  **(TEST1)** | **Slope**  **(TEST2)** | |
| LR | 0.041 (0.037–0.046) | 0.039 (0.035–0.042) | 0.275 (0.045–0.533) | -0.084 (-0.299–0.131) | | 1.044 (0.959–1.154) | 0.939 (0.869–1.021) | |
| SVM | 0.042 (0.037–0.047) | 0.040 (0.036–0.043) | 0.257 (0.031–0.501) | -0.189 (-0.422–0.023) | | 1.025 (0.935–1.133) | 0.893 (0.819–0.968) | |
| NB | 0.045 (0.040–0.050) | 0.042 (0.039–0.046) | 0.513 (0.254–0.795) | -0.849 (-1.147–-0.549) | | 1.157 (1.049–1.278) | 0.663 (0.560–0.777) | |
| NNet | 0.043 (0.039–0.049) | 0.039 (0.036–0.043) | 0.376 (0.123–0.650) | -0.176 (-0.391–0.035) | | 1.073 (0.970–1.186) | 0.951 (0.874–1.035) | |
| DT | 0.063 (0.057–0.070) | 0.044 (0.041–0.048) | 1.746 (0.216–3.229) | -0.677 (-1.502–0.521) | | 1.690 (1.044–2.362) | 0.857 (0.537–1.360) | |
| BaggedDT | 0.041 (0.036–0.046) | 0.039 (0.036–0.043) | 0.261 (0.042–0.512) | 0.242 (0.009–0.487) | | 1.092 (1.000–1.206) | 0.979 (0.904–1.057) | |
| BoostedDT | 0.040 (0.035–0.045) | 0.039 (0.035–0.042) | 0.129 (-0.112–0.354) | -0.109 (-0.312–0.146) | | 0.993 (0.907–1.092) | 0.938 (0.866–1.024) | |
| **C. 1-Year Mortality** | | | | | | | | |
| **Model** | **Brier**  **(TEST1)** | **Brier**  **(TEST2)** | **Intercept (TEST1)** | | **Intercept**  **(TEST2)** | **Slope**  **(TEST1)** | | **Slope**  **(TEST2)** |
| LR | 0.072 (0.066–0.079) | 0.071 (0.066–0.076) | 0.144 (-0.043–0.345) | | 0.184 (0.007–0.359) | 0.990 (0.914–1.077) | | 0.987 (0.916–1.061) |
| SVM | 0.073 (0.067–0.079) | 0.072 (0.067–0.076) | 0.084 (-0.092–0.279) | | 0.111 (-0.055–0.282) | 0.960 (0.885–1.038) | | 0.945 (0.878–1.014) |
| NB | 0.078 (0.071–0.084) | 0.076 (0.071–0.080) | 0.424 (0.164–0.695) | | -0.379 (-0.651–-0.097) | 1.173 (1.048–1.317) | | 0.824 (0.703–0.944) |
| NNet | 0.074 (0.067–0.080) | 0.072 (0.067–0.077) | 0.143 (-0.026–0.327) | | 0.089 (-0.087–0.269) | 1.000 (0.927–1.084) | | 0.939 (0.866–1.013) |
| DT | 0.099 (0.092–0.107) | 0.081 (0.077–0.086) | 0.589 (-0.066–1.504) | | -0.192 (-0.561–0.213) | 1.307 (0.967–1.820) | | 1.009 (0.827–1.210) |
| BaggedDT | 0.073 (0.066–0.079) | 0.072 (0.067–0.077) | 0.173 (0.012–0.353) | | 0.252 (0.078–0.416) | 1.021 (0.946–1.106) | | 0.993 (0.927–1.057) |
| BoostedDT | 0.071 (0.064–0.077) | 0.071 (0.066–0.075) | 0.048 (-0.137–0.221) | | -0.003 (-0.177–0.172) | 0.946 (0.871–1.024) | | 0.957 (0.888–1.033) |

**Table A9. Threshold-based performance on external validation cohorts (TEST1 and TEST2). Sensitivity, specificity, and Matthews correlation coefficient (MCC) are reported for (A) in-hospital, (B) 30-day, and (C) 1-year mortality. Values are estimates with 95% bootstrap confidence intervals.**

| **A. In-Hospital Mortality** | | | | | | |
| --- | --- | --- | --- | --- | --- | --- |
| **Model** | **Sensitivity**  **(TEST1)** | **Sensitivity (TEST2)** | **Specificity**  **(TEST1)** | **Specificity**  **(TEST2)** | **MCC**  **(TEST1)** | **MCC**  **(TEST2)** |
| LR | 0.872 (0.825–0.913) | 0.723 (0.670–0.775) | 0.868 (0.857–0.878) | 0.869 (0.862–0.876) | 0.446 (0.406–0.480) | 0.291 (0.259–0.319) |
| SVM | 0.855 (0.809–0.901) | 0.697 (0.640–0.753) | 0.874 (0.864–0.885) | 0.875 (0.868–0.882) | 0.447 (0.409–0.483) | 0.286 (0.255–0.319) |
| NB | 0.355 (0.295–0.418) | 0.064 (0.035–0.095) | 0.988 (0.984–0.991) | 0.998 (0.997–0.999) | 0.448 (0.383–0.513) | 0.184 (0.111–0.246) |
| NNet | 0.803 (0.752–0.855) | 0.625 (0.566–0.682) | 0.917 (0.908–0.926) | 0.924 (0.919–0.930) | 0.500 (0.455–0.543) | 0.332 (0.293–0.371) |
| DT | 0.662 (0.600–0.722) | 0.470 (0.411–0.530) | 0.892 (0.882–0.902) | 0.908 (0.902–0.914) | 0.366 (0.322–0.408) | 0.218 (0.183–0.255) |
| BaggedDT | 0.885 (0.844–0.924) | 0.621 (0.563–0.678) | 0.877 (0.868–0.886) | 0.927 (0.922–0.933) | 0.468 (0.435–0.500) | 0.336 (0.298–0.375) |
| BoostedDT | 0.786 (0.738–0.836) | 0.523 (0.457–0.579) | 0.936 (0.928–0.943) | 0.959 (0.954–0.963) | 0.540 (0.496–0.585) | 0.366 (0.318–0.409) |
| **B. 30-Day Mortality** | | | | | | |
| **Model** | **Sensitivity**  **(TEST1)** | **Sensitivity (TEST2)** | **Specificity**  **(TEST1)** | **Specificity**  **(TEST2)** | **MCC**  **(TEST1)** | **MCC**  **(TEST2)** |
| LR | 0.815 (0.771–0.856) | 0.628 (0.580–0.677) | 0.883 (0.873–0.894) | 0.876 (0.869–0.883) | 0.493 (0.456–0.529) | 0.304 (0.275–0.335) |
| SVM | 0.798 (0.754–0.843) | 0.584 (0.536–0.631) | 0.898 (0.889–0.907) | 0.894 (0.888–0.901) | 0.510 (0.472–0.549) | 0.306 (0.275–0.338) |
| NB | 0.298 (0.245–0.352) | 0.072 (0.049–0.100) | 0.993 (0.990–0.996) | 0.996 (0.994–0.997) | 0.461 (0.400–0.515) | 0.171 (0.120–0.224) |
| NNet | 0.788 (0.739–0.833) | 0.574 (0.528–0.622) | 0.901 (0.891–0.911) | 0.878 (0.871–0.885) | 0.510 (0.468–0.552) | 0.276 (0.244–0.310) |
| DT | 0.723 (0.669–0.774) | 0.486 (0.437–0.536) | 0.846 (0.833–0.857) | 0.852 (0.843–0.860) | 0.379 (0.340–0.419) | 0.196 (0.167–0.224) |
| BaggedDT | 0.808 (0.763–0.853) | 0.491 (0.442–0.543) | 0.890 (0.880–0.901) | 0.933 (0.928–0.938) | 0.502 (0.463–0.541) | 0.323 (0.283–0.361) |
| BoostedDT | 0.815 (0.769–0.857) | 0.591 (0.540–0.638) | 0.901 (0.891–0.910) | 0.897 (0.890–0.904) | 0.526 (0.486–0.564) | 0.315 (0.279–0.347) |
| **C. 1-Year Mortality** | | | | | | |
| **Model** | **Sensitivity**  **(TEST1)** | **Sensitivity (TEST2)** | **Specificity**  **(TEST1)** | **Specificity**  **(TEST2)** | **MCC**  **(TEST1)** | **MCC**  **(TEST2)** |
| LR | 0.692 (0.648–0.734) | 0.527 (0.489–0.562) | 0.860 (0.848–0.871) | 0.873 (0.867–0.881) | 0.442 (0.405–0.479) | 0.313 (0.284–0.342) |
| SVM | 0.689 (0.644–0.730) | 0.523 (0.487–0.557) | 0.860 (0.849–0.872) | 0.884 (0.877–0.891) | 0.441 (0.401–0.480) | 0.326 (0.296–0.353) |
| NB | 0.227 (0.188–0.262) | 0.064 (0.047–0.082) | 0.990 (0.986–0.993) | 0.994 (0.992–0.996) | 0.377 (0.326–0.423) | 0.160 (0.119–0.198) |
| NNet | 0.669 (0.625–0.713) | 0.510 (0.472–0.546) | 0.867 (0.854–0.879) | 0.876 (0.868–0.883) | 0.437 (0.397–0.474) | 0.305 (0.276–0.332) |
| DT | 0.644 (0.600–0.688) | 0.501 (0.467–0.534) | 0.779 (0.765–0.792) | 0.797 (0.789–0.807) | 0.308 (0.271–0.343) | 0.205 (0.180–0.229) |
| BaggedDT | 0.685 (0.640–0.726) | 0.526 (0.488–0.561) | 0.849 (0.837–0.862) | 0.893 (0.886–0.900) | 0.422 (0.387–0.460) | 0.344 (0.314–0.374) |
| BoostedDT | 0.685 (0.641–0.730) | 0.569 (0.530–0.603) | 0.870 (0.858–0.882) | 0.857 (0.849–0.865) | 0.453 (0.414–0.494) | 0.319 (0.289–0.344) |

**Table A10. Cross-model stable predictors for in-hospital, 30-day, and 1-year mortality.
Predictors are clinical-variable blocks ranked by block permutation importance (mean ROC-AUC decrease). Shown are predictors appearing in the top 30 of ≥5/7 models. Median rank and IQR summarise cross-model stability; n/N indicates the number of models in which the predictor was in the top 30 (N=7).**

| **A. In-Hospital Mortality** |  |  |  |
| --- | --- | --- | --- |
| **Predictor** | **Median Rank Across Models** | **IQR of Rank** | **Models in Top 30 (n/N)** |
| 1. Killip class on presentation | 2 | 0.75 | 7/7 |
| 1. Heart rate | 3 | 1 | 6/7 |
| 1. Age at notification | 3 | 3.25 | 7/7 |
| 1. Renal function: MDRD | 4 | 1.5 | 7/7 |
| 1. Systolic blood pressure | 6 | 3.5 | 7/7 |
| 1. Percutaneous entry: femoral | 8 | 6.25 | 7/7 |
| 1. Diastolic blood pressure | 9 | 6.75 | 7/7 |
| 1. PCI status | 9 | 6 | 6/7 |
| 1. NYHA functional classification | 11 | 7.25 | 7/7 |
| 1. Planned DAPT duration | 11 | 4 | 6/7 |
| 1. PCI strategy for STEMI | 12 | 6 | 6/7 |
| 1. Smoking status | 14 | 19 | 5/7 |
| 1. Final lesion result | 17 | 10 | 5/7 |
| 1. Post-intervention TIMI flow | 21 | 13.8 | 5/7 |
| 1. LMS lesion treated | 22 | 10 | 5/7 |
| 1. Ethnicity | 23 | 13 | 5/7 |
| 1. Percutaneous entry: radial | 23 | 26.3 | 5/7 |
| 1. Chronic renal failure | 24 | 15.5 | 5/7 |
| 1. Number of stents implanted | 24 | 26.5 | 5/7 |
| 1. Fondaparinux use | 28 | 17 | 5/7 |
| **B. 30-Day Mortality** | | | |
| **Predictor** | **Median Rank Across Models** | **IQR of Rank** | **Models in Top 30 (n/N)** |
| 1. Age at notification | 1 | 5.75 | 7/7 |
| 1. Heart rate | 2 | 0.75 | 7/7 |
| 1. Systolic blood pressure | 3 | 0.75 | 7/7 |
| 1. Killip class on presentation | 4 | 2.5 | 7/7 |
| 1. Renal function: MDRD | 5 | 1 | 7/7 |
| 1. Percutaneous entry: femoral | 9 | 6.25 | 7/7 |
| 1. PCI strategy for STEMI | 9 | 12 | 5/7 |
| 1. PCI status | 13 | 8 | 6/7 |
| 1. NYHA functional classification | 13 | 8 | 7/7 |
| 1. Planned DAPT duration | 15 | 10 | 6/7 |
| 1. LMS lesion treated | 17 | 20.8 | 5/7 |
| 1. Post-intervention TIMI flow | 18 | 16.8 | 5/7 |
| 1. Baseline serum creatinine | 19 | 25.5 | 5/7 |
| 1. Fondaparinux use | 19 | 10.8 | 5/7 |
| 1. Diastolic blood pressure | 20 | 18.5 | 6/7 |
| 1. Gender | 20 | 16 | 5/7 |
| 1. CAD Presentation | 22 | 10 | 5/7 |
| 1. Number of stents implanted | 24 | 14 | 5/7 |
| 1. Drug-eluting stent | 27 | 13 | 5/7 |
| **C. 1-Year Mortality** | | | |
| **Predictor** | **Median Rank Across Models** | **IQR of Rank** | **Models in Top 30 (n/N)** |
| 1. Age at notification | 1 | 2.5 | 7/7 |
| 1. Heart rate | 2 | 1 | 7/7 |
| 1. Killip class on presentation | 3 | 1.5 | 7/7 |
| 1. Renal function: MDRD | 4 | 2 | 6/7 |
| 1. Systolic blood pressure | 6 | 1 | 7/7 |
| 1. Diastolic blood pressure | 11 | 19.3 | 6/7 |
| 1. Diabetes mellitus | 11 | 7 | 7/7 |
| 1. Percutaneous entry: femoral | 11 | 10.5 | 6/7 |
| 1. Number of stents implanted | 13 | 16 | 6/7 |
| 1. Body mass index | 14 | 21.8 | 5/7 |
| 1. Drug-eluting stent | 14 | 6.25 | 7/7 |
| 1. NYHA functional classification | 15 | 11.3 | 6/7 |
| 1. Planned DAPT duration | 16 | 3 | 6/7 |
| 1. Smoking status | 16 | 12 | 5/7 |
| 1. Post-intervention TIMI flow | 17 | 2.5 | 6/7 |
| 1. PCI strategy for STEMI | 18 | 13 | 6/7 |
| 1. Coronary disease (>50% stenosis) | 19 | 13 | 6/7 |
| 1. Dyslipidaemia | 25 | 9 | 5/7 |
| 1. Percutaneous entry: radial | 27 | 28.5 | 5/7 |
